# The roles of serine hydrolases and serum albumin in alisol B 23-acetate hydrolysis in humans

**DOI:** 10.3389/fphar.2023.1160665

**Published:** 2023-04-06

**Authors:** Tiantian Zhang, Feng Zhang, Yani Zhang, Hongxin Li, Guanghao Zhu, Taotao Weng, Cheng Huang, Ping Wang, Yuqi He, Jing Hu, Guangbo Ge

**Affiliations:** ^1^ School of Pharmacy, Zunyi Medical University, Zunyi, Guizhou, China; ^2^ Shanghai Frontiers Science Center of TCM Chemical Biology, Institute of Interdisciplinary Integrative Medicine Research, Shanghai University of Traditional Chinese Medicine, Shanghai, China; ^3^ Department of Nephrology, The Seventh People’s Hospital of Shanghai University of Traditional Chinese Medicine, Shanghai, China; ^4^ School of Chinese Medicine, Shanghai University of Traditional Chinese Medicine, Shanghai, China

**Keywords:** alisol B 23-acetate, alisol B, human serum albumin (hSA), serine hydrolases, farnesoid X receptor (FXR)

## Abstract

**Introduction:** Alisol B 23-acetate (AB23A), a major bioactive constituent in the Chinese herb Zexie (*Rhizoma Alismatis*), has been found with multiple pharmacological activities. AB23A can be readily hydrolyzed to alisol B in mammals, but the hydrolytic pathways of AB23A in humans and the key enzymes responsible for AB23A hydrolysis are still unrevealed. This study aims to reveal the metabolic organs and the crucial enzymes responsible for AB23A hydrolysis in human biological systems, as well as to decipher the impact of AB23A hydrolysis on its biological effects.

**Methods:** The hydrolytic pathways of AB23A in human plasma and tissue preparations were carefully investigated by using Q-Exactive quadrupole-Orbitrap mass spectrometer and LC-UV, while the key enzymes responsible for AB23A hydrolysis were studied via performing a set of assays including reaction phenotyping assays, chemical inhibition assays, and enzyme kinetics analyses. Finally, the agonist effects of both AB23A and its hydrolytic metabolite(s) on FXR were tested at the cellular level.

**Results:** AB23A could be readily hydrolyzed to form alisol B in human plasma, intestinal and hepatic preparations, while human butyrylcholinesterase (hBchE) and human carboxylesterases played key roles in AB23A hydrolysis in human plasma and tissue preparations, respectively. It was also found that human serum albumin (hSA) could catalyze AB23A hydrolysis, while multiple lysine residues of hSA were covalently modified by AB23A, suggesting that hSA catalyzed AB23A hydrolysis *via* its pseudo-esterase activity. Biological tests revealed that both AB23A and alisol B exhibited similar FXR agonist effects, indicating AB23A hydrolysis did not affect its FXR agonist effect.

**Discussion:** This study deciphers the hydrolytic pathways of AB23A in human biological systems, which is very helpful for deep understanding of the metabolic rates of AB23A in humans, and useful for developing novel prodrugs of alisol B with desirable pharmacokinetic behaviors.

## 1 Introduction

*Rhizoma Alismatis* (also named Zexie in Chinese), a famous Chinese herb, is widely used in clinical settings for treating a range of human diseases (Jia et al., 2022; Yan et al., 2022). As a key herbal material, *Rhizoma Alismatis* is used for the preparation of a variety of Chinese herbal prescriptions, such as Zexie Decoction, Liuwei Dihuang Pill, Longdan Xiegan Pill, Zhuling Decoction, Zhibai Dihuang pill, and Qingfei Paidu Decoction (Ye et al., 2009; Liu et al., 2011; Dai et al., 2020; Wu J. et al., 2021; Ma et al., 2021; Zhang et al., 2021). Increasing modern pharmacological evidence has shown that *Rhizoma Alismatis* has multiple biological activities, including diuresis, anti-nephritis, anti-atherosclerosis, anti-inflammation, anti-tumor, immune regulatory effect, hepatoprotective effect, blood-lipid and blood-glucose lowering activities (Li et al., 2017; Meng et al., 2017; Zhang et al., 2017; Liu S. S. et al., 2019; Cheng et al., 2019; Li et al., 2020; Xu et al., 2020; Ye et al., 2022). Previous studies have suggested that *Rhizoma Alismatis* contains a variety of constituents including terpenoids, sterols, glycosides, and flavonoids, while triterpenoids have been reported as the major active constituents (Liu et al., 2020; Bailly C., 2022). Among all reported terpenoids in *Rhizoma Alismatis*, Alisol B 23-acetate has drawn much attention in the past few decades, owing to its high abundance and broad pharmacological activities (Kwon et al., 2021; Sun et al., 2021). Consequently, the Chinese Pharmacopoeia of the 2020 edition has recommended that alisol B 23-acetate (AB23A) and alisol C 23-acetate should be used as the maker compounds for quality control of *Rhizoma Alismatis*.

As one of the major bioactive constituents in *Rhizoma Alismatis*, the pharmacological activities of AB23A have been extensively investigated in the past few decades. Previous studies have showed that AB23A is a naturally occurring farnesoid X receptor (FXR) agonist, while this agent can ameliorate renal ischemia-reperfusion injury and promote liver regeneration *via* activating FXR (Meng et al., 2014; Luan et al., 2021). Furthermore, AB23A can relieve dyslipidemia and inflammation in atherosclerosis, inhibit mast cell activation, reverse p-glycoprotein-mediated multidrug resistance, modulate the renin-angiotensin system and attenuate chronic kidney disease (Sun et al., 2021; Chen et al., 2020). In addition, many studies have reported that AB23A has the potential to be a novel anticancer agent for the treatment of various types of cancer, such as hepatocellular carcinoma, gastric cancer, lung cancer, and ovarian cancer (Li et al., 2020; Kwon et al., 2021; Xia et al., 2019; Liu Y. et al., 2019; Zhang et al., 2016; Zhu et al., 2021). Although the pharmacological effects of AB23A have been extensively studied in the past few decades, the metabolism and pharmacokinetic studies of AB23A are rarely reported. Especially, the major metabolic pathways and the key enzymes responsible for AB23A metabolism in humans are still unclear.

Previous studies have reported that the content of AB23A is significantly higher than that of alisol B in *Rhizoma Alismatis* and its related herbal products (Wu et al., 2022). However, recent studies have found that AB23A can be readily hydrolyzed to release alisol B (AB) in rats, while AB can further undergo cytochrome P450 enzymes (CYPs) mediated oxidative metabolism (Tao et al., 2019). More recently, Liu et al. (2022) have reported that AB23A is hardly detected in mice plasma and liver after oral administration of Qingfei Paidu Decoction (a marketed Chinese herbal medicine containing *Rhizoma Alismatis*), but AB and its oxidative metabolite could be readily detected in mice plasma and liver after oral administration of this herbal medicine. Our preliminary study showed that AB23A could be readily hydrolyzed to release AB in the liver microsomes from a panel of animal species (Supplementary Figures S1, S4; Supplementary Table S1). The above findings demonstrated that AB23A could be easily hydrolyzed to AB both *in vitro* and *in vivo*. However, the major metabolic organs and enzymes responsible for AB23A hydrolysis, as well as the impact of AB23A hydrolysis on its biological effects are still unrevealed.

**FIGURE 1:**
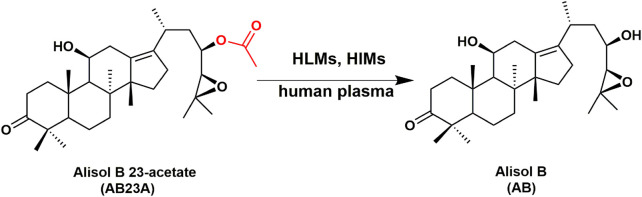
The hydrolytic pathways of AB23A in HLMs, HIMs and human plasma.

**FIGURE 2:**
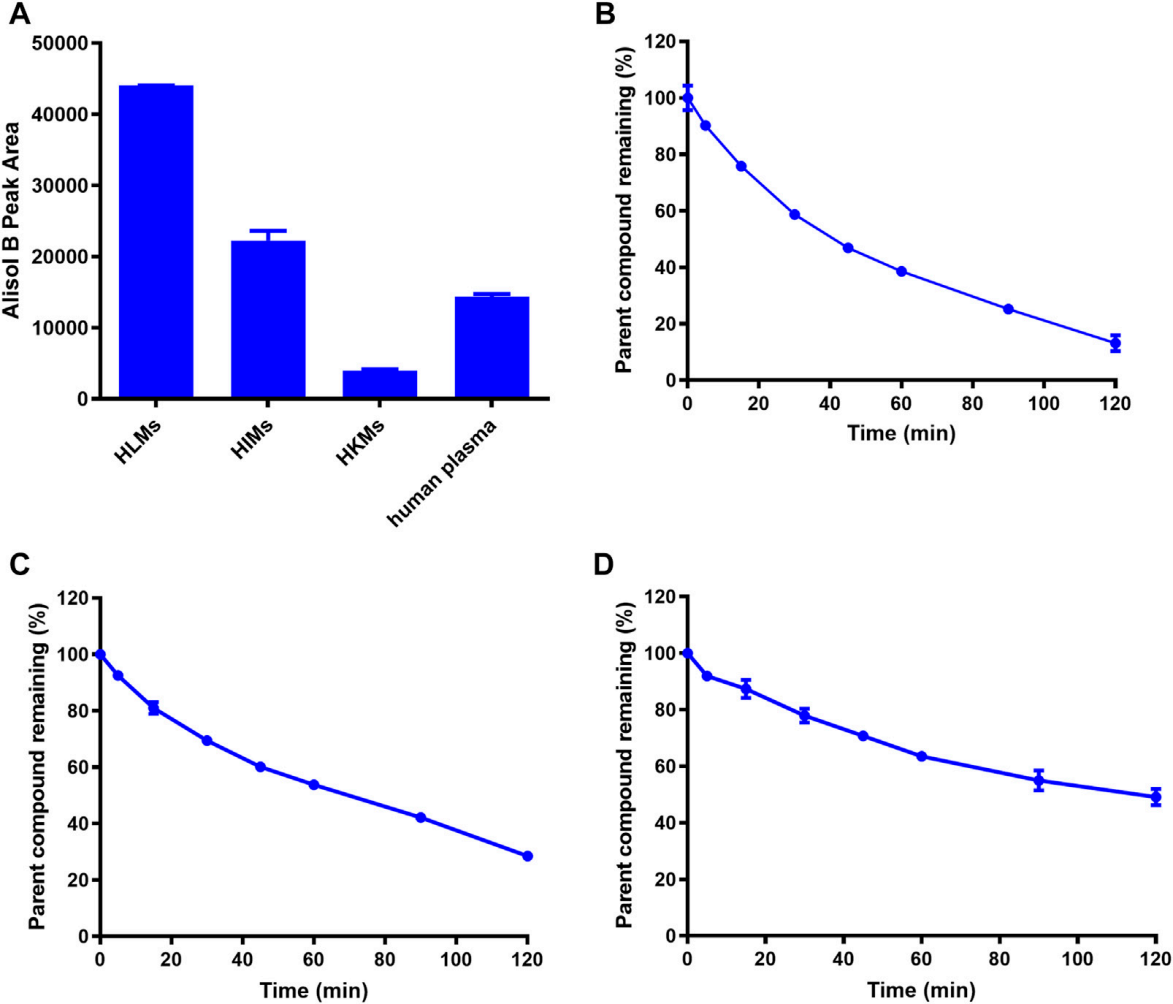
The metabolic organs **(A)** and hydrolytic half-lives (t_1/2_) of AB23A in HLMs **(B)**, HIMs **(C)**, human plasma (25% dilution) **(D)**. The data were expressed as mean ± SD.

This research aims to solve the following two key questions involved in AB23A hydrolysis, 1) to reveal the major metabolic organs and the key enzymes responsible for AB23A hydrolysis in human biological systems (Figure 1), 2) to characterize the FXR agonist effects of both AB23A and its hydrolytic metabolite AB. For these purposes, the hydrolytic metabolite profiles and metabolic stabilities of AB23A in human plasma and tissue preparations were carefully investigated, while the key enzymes responsible for AB23A hydrolysis in human biological systems were assigned *via* performing a set of assays including reaction phenotyping assays, chemical inhibition assays, and enzyme kinetics analyses. Finally, the agonist effects of both AB23A and its hydrolytic metabolite(s) on FXR were tested at the cellular level, to reveal the impact of AB23A hydrolysis on its biological activity.

## 2 Materials and methods

### 2.1 Chemicals and reagents

Alisol B 23-acetate (AB23A) and alisol B (AB) were ordered from Chengdu preferred Biological Technology Co., Ltd., (Chengdu, China). Loperamide (LPA) and bis(p-nitrophenyl) phosphate (BNPP) were ordered from TCI Development Co., Ltd., (Shanghai, China). Ethylenediaminetetraacetic acid (EDTA) and galanthamine (GA) were produced from J&K Chemical Co., Ltd., (Beijing, China). Boceprevir was purchased from Dalian Meilun Biotechnology Co., Ltd., (Dalian China). GW4064 was obtained from Sigma-Aldrich (MO, United States). The ultrapure water purified by Milli-Q^®^ Integral Water Purification System (Millipore, United States). Methanol (LC grade) and acetonitrile (LC grade) were provided from Fisher Scientific Co., Ltd., (Fair Lawn, NJ, United States), while formic acid (LC grade) was supplied from Shanghai Aladdin Biochemical Technology Co., Ltd., (Shanghai, China). FuGENE-HD and HEK293T cells were obtained by Roche and ATCC, respectively.

### 2.2 Enzymes and tissue preparations

The pooled human liver microsomes from 50 donors (HLMs, Lot No. 2010065) and pooled human Intestine microsomes from 6 donors (HIMs, Lot No. 1410066) were supplied from XenoTech (United States). Human kidney microsomes (HKMs, Lot No. X03801), pig liver microsomes (PLMs), rat liver microsomes (RLMs), mouse liver microsomes (MLMs) and human plasma were provided by Research Institute for Liver Diseases (Shanghai) Co. Ltd., (RILD, Shanghai, China). Human acetylcholinesterase (hAchE, Lot No. C1682), human butyrylcholinesterase (hBchE, Lot SLCG5158), and human serum albumin (hSA, Lot#SLCC9268) were provided by Sigma-Aldrich (MO, United States). Human monoacylglycerol lipase (hMAGL, Lot No. 10007812) was purchased from Cayman Chemicals (Ann Arbor, Michigan, United States) and human cathepsin A (hCTSA, Lot No. FXY0619021) was purchased from R&D systems Co., Ltd., (Minnesota, United States). Human carboxylesterase 1A (hCES1A), human carboxylesterase 2A (hCES2A), human Notum (hNotum), human pancreatic lipase (hPL), and human isoamyl acetate-hydrolyzing esterase 1 (hIAH1, unpublished) were expressed and purified as described previously (Morton and Potter, 2000; Lamego et al., 2013; Hu et al., 2021). All biological reagents were stored at −80°C until use.

### 2.3 Metabolite profiling of AB23A and its hydrolytic metabolite

AB23A and its hydrolytic metabolite were separated by ACQUITY UPLC I-Class plus system (Waters Corporation, Milford, United States) coupled with ACQUITY UPLC HSS T3 (100 mm × 2.1 mm, 1.8 μm, Waters, United States), and identified by using Q-Exactive quadru-pole-Orbitrap mass spectrometer. The mobile phases consisted of 0.1% formic acid in water (A) and acetonitrile (B) with the elution gradients were as follows: 0–2 min, 5% B, 2–4 min, 5%–30% B, 4–8 min, 30%–50% B, 8–10 min, 50%–80% B, 10–14 min, 80%–100%, 14–15 min, 100% B, 15–15.1 min, 100%–5% B, 15.1–16 min, 5% B. The flow rate was set at 0.35 mL/min, while the temperature of chromatographic column was maintained at 45°C. The mass range was set from *m/z* 80 to 1,200. Other MS conditions were as follows: Resolution and AGC target were set at 30000 and 1e^6^, the maximum IT was 50 ms and loop count was set 10. (N) CE/stepped (N)CE was set 10, 20, 40 ev. All data were analyzed by PeakView 2.2 software and Thermo Xcalibur.

### 2.4 The hydrolytic half-lives of AB23A in human plasma and tissue preparations

The hydrolytic half-lives (t_1/2_) of AB23A in human plasma and different human tissue preparations were carried out according to the procedure of *in vitro* metabolic stability studies (Ge et al., 2013). The incubation mixture (1,000 μL) consisted of PBS (pH 7.4), enzyme sources (HLMs/HIMs/human plasma) and AB23A. 25% diluted human plasma was used, the protein concentrations of other tissue microsomes were 0.1 mg/mL. The 100 μL incubation mixture was moved to another containing ice-cold acetonitrile tube at different time points (0, 5, 15, 30, 45, 60, 90, 120 min). All samples were centrifuged for 30 min at 20,000 g 4°C and took the supernatants for LC-UV analysis.

### 2.5 Reaction phenotyping assays

The hydrolytic rates of AB23A in different human recombinant enzymes (hCES1A, hCES2A, hPL, hIAH1, hNotum, hAchE, hBchE, hSA, hCTSA, hMAGL) were assayed. The incubation system included PBS, each tested enzyme (5 μg/mL, final concentration), and AB23A (20 μM). After incubation for 1 h at 37°C, ice-cold acetonitrile was added to terminate the reaction and precipitate proteins. The supernatants by centrifuging were used to quantify the formation rates of AB by LC-UV analysis. The detection wavelength of AB was 196 nm.

### 2.6 Chemical inhibition assays

The inhibitory effects of a series of selective inhibitors of human hydrolases against AB23A hydrolysis were assessed. The selective inhibitors of human hydrolases including BNPP (50 μM, a specific inactivator of CES), boceprevir (20 μM, a potent inhibitor for CTSA), LPA (100 μM, a selective inhibitor for CES2A), GA (100 μM, a cholinesterase inhibitor), EDTA (20 μM, a selective PONs inhibitor). The incubation system included PBS, inhibitors, HLMs (0.1 mg/mL)/human plasma (25% dilution), and AB23A (20 μM). After reacting for 1 h, ice-cold acetonitrile was used to terminate the reaction. The supernatants by centrifuging were used to quantify the formation rates of AB by LC-UV analysis.

### 2.7 Kinetic analyses of AB23A hydrolysis in different enzyme sources

The enzymatic kinetics of AB23A hydrolysis in different tissue preparations (HLMs, HIMs, human plasma) and human recombinant enzymes (hCES1A, hCES2A, hSA, hBchE) were analyzed. The incubation system (200 μL) consisted of PBS, each tested enzyme, and AB23A (ranging from 0 to 1,000 μM). After incubating for 1 h, the supernatants were then used for LC-UV analysis. The kinetic parameters of AB23A hydrolysis were fitted by the following Michaelis−Menten Eq. 1 (Zhang et al., 2022).

$$V=\left(V_{\max}\times \left[S\right]\right)⁄\left(K_{m}+\left[S\right]\right)$$ (1)

Here, *K*_*m*_ is the Michaelis constant of AB23A hydrolysis, *V*_*max*_ is the maximum velocity of AB23A hydrolysis, *V*_*max*_/*K*_*m*_ is used to calculate the intrinsic clearance (*CL*_*int*_).

### 2.8 Identification of the modification sites of hSA by AB23A

Human serum albumin (1 mg/mL) and AB23A (50 μM) were co-incubated at 37°C for 2 h and then transferred to 10 kDa centrifugal filter tubes. The soluble impurities and unbound molecules were washed by ultrapure water (200 μL) (Nel et al., 2015). After denaturation for 1 h in 8 M urea solution at 37°C, the mixture was alkylated with dithiothreitol and iodoacetamide in the dark, washed twice with 200 μL ultrapure water, and finally washed with 200 μL 50 mM ammonium bicarbonate. The samples were digested overnight with 2 μL trypsin in 50 mM ammonium bicarbonate solution at an enzyme-to-substrate ratio of 1:50 (w/w). The trypsin digest was collected and washed with 50 μL NaCl solution (500 mM). The peptides were desalted by the MonoSpin C18 (GL Sciences). Subsequently, the eluents were dried by the vacuum pump and resolved in 40 μL 0.1% formic acid for analyses (please refer to the Supplementary Material for more details).

### 2.9 FXR agonist activities of AB23A and AB

The FXR agonist activities were performed by the Dual-Luciferase Reporter Assay System as the previously reported scheme (Huang et al., 2006). The phFXR, phRXR (retinoid X receptor) and FXR-dependent reporter (EcRE-LUC) were expressed and cotransfected with the Gal4 reporter vector MH100 × 4-TK-Luc, then 1 μg of the relevant plasmid was combined with 1 μg of reporter plasmids and 0.1 μg of pREP7 (Renilla Luciferase) reporter, which was used to normalize transfection efficiencies. HEK293T cells were dispensed into 96 well plates using a volume of 100 μL and 1 × 10^5^ cells per well. The transfection mixture (contained 10 μg of total plasmids and 15 μL FuGENE-HD per ml of DMEM was added to HEK293T cells. When the confluence of cells achieved 50%, a transfection mixture was configured in serum-free DMEM. After 4–6 h, the medium was removed. AB, AB23A and positive agonist (GW4064) were added to each well using a volume of 100 µL and incubated for 48 h. The promoter activities were determined by the Steady-Glo reagent. GW4064 was used as the positive agonist, which maximum efficacy was set to 100%.

### 2.10 Molecular docking simulations

The X-ray crystal structures of hCES1A1 (PDB ID 2dr0), hAchE (PDB ID 4ey7), hBchE (PDB ID 1p0i) were used for docking simulations, while the 3D structure of hCES2A was obtained by homology modeling described previously. The MM2 force field in ChemBio 3D Ultra 15.0 was used to obtain the energy-minimized conformation of compound. The proteins and ligands were prepared using AutoDock Vina (1.1.2), including adjusting polar hydrogen atoms only, atomic charges as Kollman, and atom types as AD4. Molecular docking simulations were performed using AutoDock Vina 1.1.2 in a grid covering the entire active site. Discovery Studio Visualizer further analyzed the binding modes, and the catalytic conformation was depicted in PyMol (Version 2.3, Schrödinger, LLC, New York City, United States).

### 2.11 Data analysis

All data were shown as mean ± SD from triplicates assays and the statistical differences were analyzed by one-way ANOVA. The *K*_*m*_ and *V*_*max*_ values were fitted by GraphPad Prism 8.0.1. The ChemDraw software was used to draw the structures.

## 3 Results

### 3.1 Identification of the hydrolytic metabolite(s) of AB23A

The hydrolytic metabolite(s) of AB23A in human plasma and tissue preparations were identified by using Q-Exactive quadrupole-Orbitrap mass spectrometer. As shown in Supplementary Figure S1, AB23A was very stable in PBS under physiological conditions (pH 7.4 at 37°C). By contrast, a polar metabolite could be detected following incubation with AB23A in human plasma, HLMs or HIMs. The polar metabolite in positive ion mode [(M + H)^+^] was *m/z* 473.43, 42 Da less than the prototype AB23A [*m/z* 515.45 Da, (M + H)^+^], suggesting that the polar metabolite was a deacetylated product of AB23A. This deacetylated product was further identified as alisol B, by comparing with the fragmentation ions and retention time of this product peak to the standard alisol B (Supplementary Figures S2, S3). These findings clearly manifested that AB23A could be readily hydrolyzed to release alisol B in human plasma and human tissue preparations.

### 3.2 The hydrolytic half-lives (t_1/2_) of AB23A in human tissue preparations and plasma

Next, the hydrolytic half-lives (t_1/2_) of AB23A were determined in human plasma and human tissue preparations (including HLMs, HIMs and HKMs). As shown in Figure 2A, AB23A could be easily hydrolyzed to release AB in HLMs, HIMs and human plasma, but only a trace amount of AB (the hydrolytic metabolite of AB23A) was detected in HKMs. The metabolic half-lives (t_1/2_) of AB23A in HLMs and HIMs were fitted as 41.00 and 68.00 min, respectively. By contrast, the metabolic half-life (t_1/2_) of AB23A in 25% diluted human plasma (114.95 min) was relatively longer than that in human tissue preparations (Figures 2B-D, Table 1). The above results demonstrated that human intestine, liver and human plasma were major organs responsible for AB23A hydrolysis in humans, which encouraged us to further investigate the key enzymes responsible for AB23A hydrolysis in human biological systems.

**TABLE 1:** *In vitro* metabolic half-lives of AB23A in HLMs, HIMs, and human plasma.

Enzyme source	Final concentration	t_1/2_ (min)
HLMs	0.1 mg/mL	41.00 ± 0.01
HIMs	0.1 mg/mL	68.00 ± 0.01
Human plasma	25% dilution plasma	114.95 ± 0.2

### 3.3 The roles of human hydrolases in AB23A hydrolysis

**FIGURE 3:**
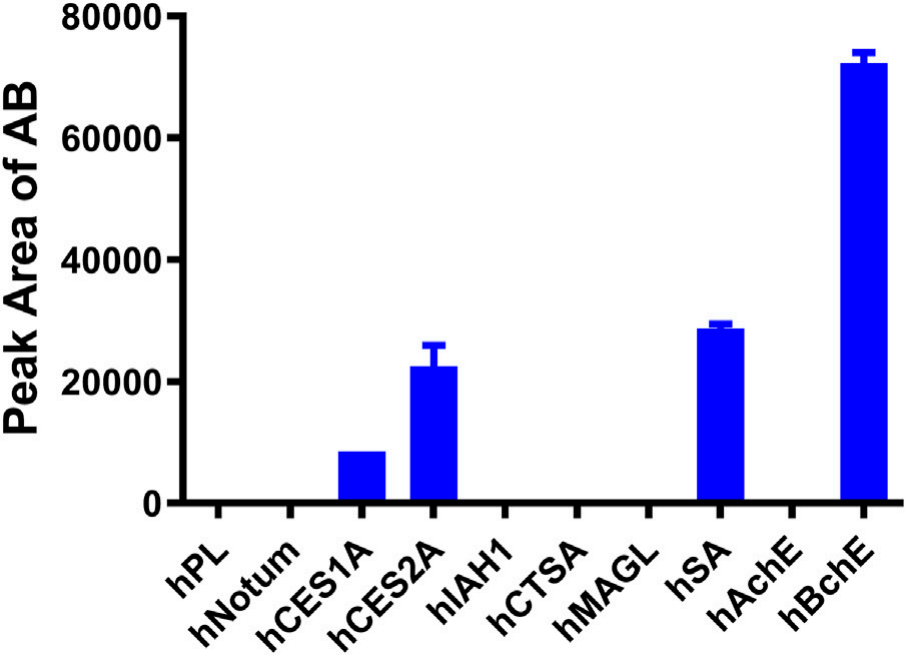
Reaction phenotyping assays of AB23A hydrolysis: AB23A (20 μM) was incubated with each tested human hydrolase for 60 min under physiological conditions (pH 7.4 at 37°C). The protein concentration of hPL, hNotum, hCES1A, hCES2A, hIAH1, hCTSA, hMAGL, hAchE, and hBchE in the reaction system were 5 μg/mL, the hSA was 500 μg/mL.

Subsequently, the key enzymes responsible for AB23A hydrolysis were studied by using reaction phenotyping assays and chemical inhibition assays. Firstly, the formation rates of the AB were determined in different human enzymes. As shown in Figure 3, hBchE showed the faster hydrolytic rate for AB23A, while hSA, hCES2A and hCES1A also catalyzed this hydrolytic reaction. Secondly, a series of specific inhibitors of human esterases were used to determine the contribution of various human esterases in AB23A hydrolysis. As shown in Supplementary Figure S5, GA (a cholinesterase inhibitor) could significantly inhibit the formation of AB in human plasma, while boceprevir and EDTA showed negligible effects on AB23A hydrolysis in human plasma. These results suggested that hBchE played an important role in AB23A hydrolysis in human plasma.

In human liver preparations (Supplementary Figure S6A), BNPP could completely blocked AB23A hydrolysis in HLMs, while LPA and GA could slightly blocked AB23A hydrolysis in HLMs. By contrast, boceprevir and EDTA showed negligible effects on AB23A hydrolysis in HLMs. Similarly, BNPP also near-completely inhibited the formation of AB in HIMs (Supplementary Figure S6B), while LPA could partially inhibit AB23A hydrolysis in HIMs. These results suggested that hCES played important roles in AB23A hydrolysis in HLMs and HIMs, while hBchE also contributed slightly to this reaction in human liver.

### 3.4 Enzymatic kinetic analyses of AB23A hydrolysis

**FIGURE 4:**
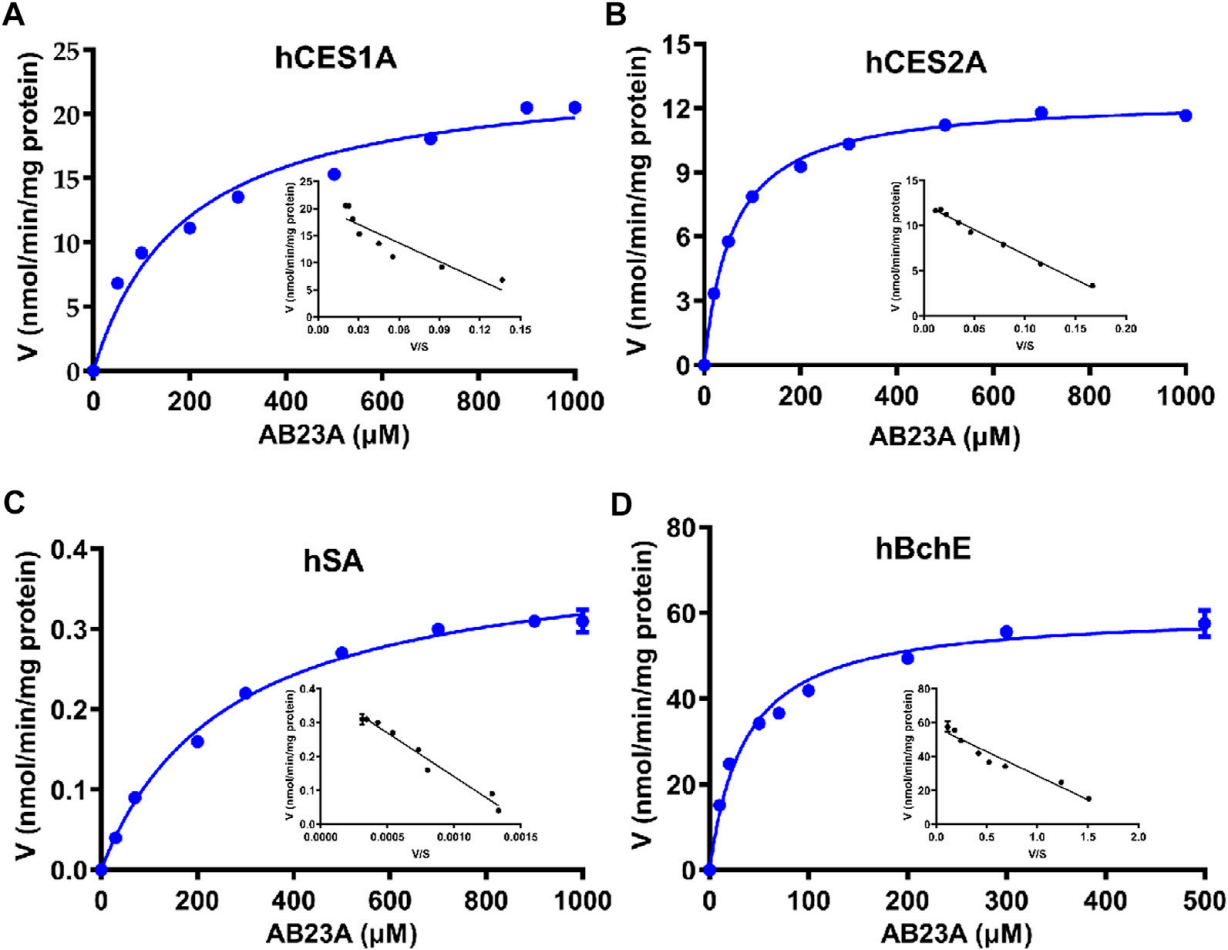
Michaelis−Menten kinetic plots of AB23A hydrolysis in hCES1A **(A)** hCES2A **(B)**, hSA **(C)** and hBchE **(D)**. The kinetic experiments were conducted in triplicate, and the data were expressed as mean ± SD.

To deeply understand the contribution of human serine hydrolases and hSA in AB23A hydrolysis in various organs, the kinetic behaviors of AB23A hydrolysis and related kinetic parameters (*K*_*m*_, *V*_*max*_, and *CL*_*int*_) were characterized in various enzyme sources including a panel of human serine hydrolases (hCES1A, hCES2A, and hBchE) and hSA, as well as human plasma (25% dilution) and tissue preparations. As shown in Table 2 and Figure 4, the Michaelis constant (*K*_*m*_) values of AB23A hydrolysis in hCES1A, hCES2A, hSA and hBchE were determined as 190.80 ± 31.41 μM, 58.96 ± 2.41 μM, 261.20 ± 18.69 μM, and 35.78 ± 3.40 μM, respectively. The maximum velocity (*V*_*max*_) values of AB23A hydrolysis in hCES1A, hCES2A, hSA and hBchE were 23.47 ± 1.10 nmol/min/mg protein, 12.47 ± 0.12 nmol/min/mg protein, 0.40 ± 0.01 nmol/min/mg protein, 60.24 ± 1.53 nmol/min/mg protein, respectively (Table 2). Consequently, the intrinsic clearance (*CL*_*int*_) values of AB23A hydrolysis in hCES1A, hCES2A, hSA and hBchE were 123.01 μL/min/mg protein, 211.50 μL/min/mg protein, 1.53 μL/min/mg protein, 1683.62 μL/min/mg protein, respectively (Table 2). These data suggest that hBchE shows the highest turnover rate for AB23A hydrolysis, while hCES2A and hCES1A also display relatively high turnover rates for this reaction. By contrast, the turnover rate of hSA for AB23A hydrolysis is relatively limited.

**TABLE 2:** Kinetic parameters of AB23A hydrolysis in hCES1A, hCES2A, hSA and hBchE.

Enzyme source	*K*_*m*_ (μM)	*V*_*max*_ (nmol/min/mg protein)	*CL*_*int*_ (μL/min/mg protein)
hCES1A	190.80 ± 31.41	23.47 ± 1.10	123.01
hCES2A	58.96 ± 2.41	12.47 ± 0.12	211.50
hBchE	35.78 ± 3.40	60.24 ± 1.53	1683.62
hSA	261.20 ± 18.69	0.40 ± 0.01	1.53

**FIGURE 5:**
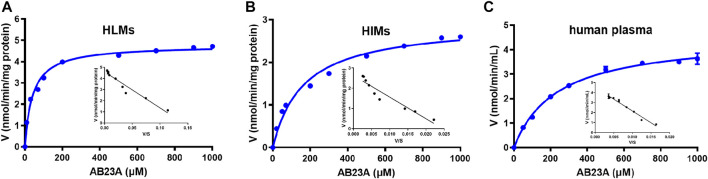
Michaelis−Menten kinetic plots of AB23A hydrolysis in HLMs **(A)**, HIMs **(B)**, and human plasma (25% dilution) **(C)**. The experiments were conducted in triplicate, while the data were expressed as mean ± SD.

As shown in Table 3 and Figure 5, AB23A hydrolysis also followed Michaelis-Menten kinetics in HLMs, HIMs, and human plasma. The *K*_*m*_ values of AB23A hydrolysis in HLMs, HIMs, and human plasma were 41.18 ± 3.46 μM, 164.00 ± 18.63 μM, and 237.10 ± 15.00 μM, respectively. The *V*_*max*_ values of AB23A hydrolysis in HLMs, HIMs and human plasma were 4.77 ± 0.08 nmol/min/mg protein, 2.93 ± 0.10 nmol/min/mg protein and 4.536 ± 0.09 nmol/min/mL, respectively. Meanwhile, the *CL*_*int*_ of AB23A hydrolysis in HLMs, HIMs and human plasma (25% dilution) were estimated as 115.93 μL/min/mg protein, 17.89 μL/min/mg protein, 19.13 μL/min/mL, respectively. Considering the high *CL*_*int*_ values of AB23A hydrolysis in human liver preparations and the large volume of human plasma (∼6 L), it is conceivable that the liver and blood should be the major organs responsible for AB23A hydrolysis in humans.

**TABLE 3:** Kinetic parameters of AB23A hydrolysis in HLMs, HIMs and human plasma.

Enzyme source	*K*_*m*_ (μM)	*V*_*max*_	*CL*_*int*_
HLMs	41.18 ± 3.46	4.77 ± 0.08^a^	115.93^c^
HIMs	164.00 ± 18.63	2.93 ± 0.10^a^	17.89^c^
Human plasma^e^	237.10 ± 15.00	4.536 ± 0.09^b^	19.13^d^

^a^ V_max_ was in nmol/min/mg protein.

^b^ V_max_ was in nmol/min/mL.

^c^ CL_int_, was in μL/min/mg protein.

^d^ CL_int_, was in μL/min/mL.

^e^ 25% dilution for human plasma.

### 3.5 hSA catalyzes AB23A hydrolysis *via* its pseudo-esterase activity

It is well-known that hSA is not an esterase but functions as a pseudo-esterase, the acyl of some ester-bearing substrates (such as aspirin and Boc 5) can covalently bind to the lysines of hSA during the hydrolytic process (Liyasova et al., 2010; Ge et al., 2013). The pseudo-esterase activity of hSA is a unique character for this abundant plasma protein, which has been used for constructing a series of specifically enzymatic activatable probes for hSA (Ge et al., 2017; Jin et al., 2017). Herein, to clarify the unique hydrolytic mode of AB23A catalyzed by hSA, the peptides of AB23A modified by hSA were fully characterized by mass spectrometry. Following optimization of analytical conditions, the peptide coverage rate of hSA was up to 96%. The lysine modification profiling showed that a total of 39 lysines in hSA could be acylated by AB23A (Supplementary Table S2), following 12 h co-incubation. All modified peptides and the modified lysine sites were listed in Supplementary Table S2, and the corresponding MS/MS spectra were shown in Supplementary Figure S8. These results clearly demonstrated that multiple lysine residues of hSA could be acetylated by AB23A, suggesting that hSA catalyzed AB23A hydrolysis *via* its unique pseudo-esterase activity.

### 3.6 Agonist activities of AB23A and AB on FXR

**FIGURE 6:**
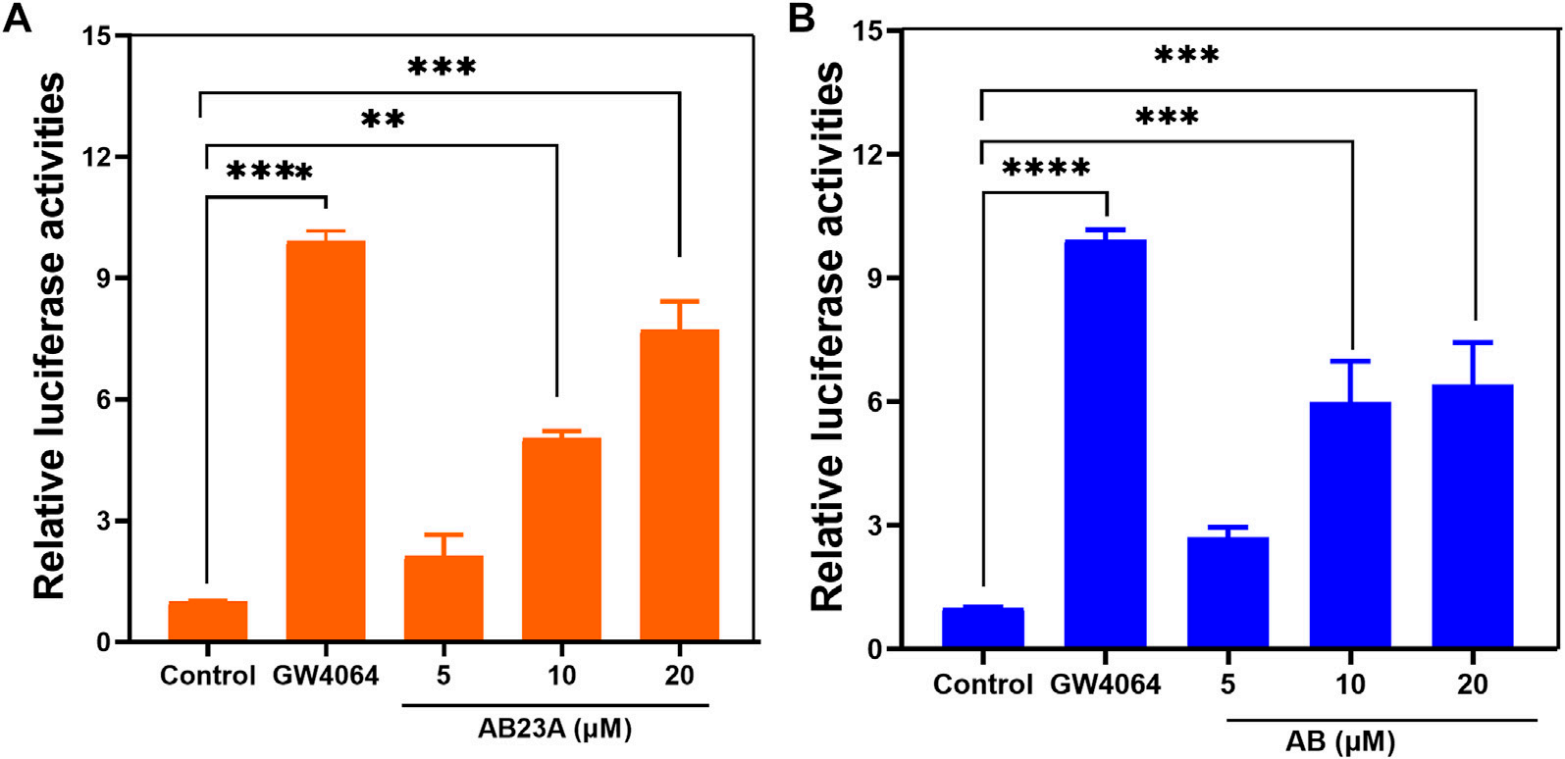
The transcriptional activation assays of AB23A **(A)** and AB **(B)**. HEK293T cells were cotransfected with phFXR expression plasmid, and FXR-dependent reporter (EcRE-LUC) and treated with the FXR agonist GW4064 (10 μM), control (DMSO), AB23A and AB (5, 10, 20 μM) for another 48 hours. The relative luciferase activities were measured by comparison with renilla luciferase activities. The maximum efficacy of the positive agonist (GW4064) is arbitrarily set at 100%. Compared with the control group, ****p < 0.0001 ***p < 0.001, **p < 0.01.

It has been reported that AB23A is a naturally occurring FXR agonist, and this compound has been validated as an efficacious agent for ameliorating renal ischemia-reperfusion injury, estrogen-induced cholestatic liver injury and liver regeneration (Meng et al., 2014; Meng et al., 2015; Luan et al., 2021). In this study, the FXR transcription transactivation activities of both AB23A and AB were tested by using HEK293T cells co-transfected with the phFXR and an FXR-dependent reporter (EcRE-LUC), while GW4064 was used as the positive agonist. As shown in Figure 6, the studies demonstrated that both AB23A and its hydrolytic metabolite AB could dose-dependently agonize FXR. Interestingly, it was seemed that AB was slightly more potent than AB23A in activating FXR at low doses (5 and 10 μM). These results demonstrate that AB has similar FXR agonist effect to AB23A, suggesting that AB23A hydrolysis do not affect its FXR agonist effect *in vivo*.

### 3.7 Molecular docking simulations

**FIGURE 7:**
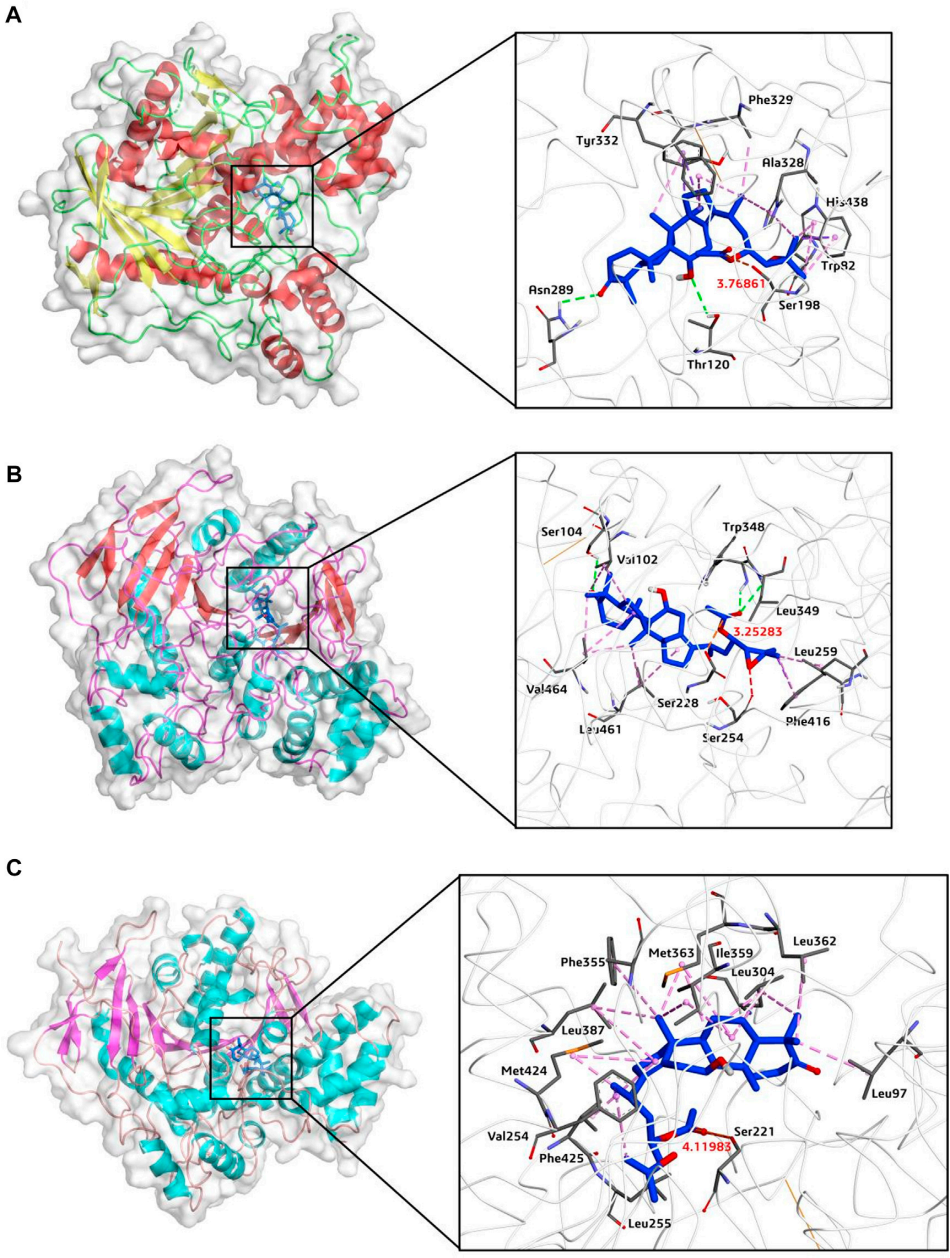
Overall binding modes and receptor-ligand interaction analysis of AB23A (blue) in hBchE **(A)**, hCES2A **(B)** and hCES1A **(C)**.

Finally, to reveal the interaction mechanisms of AB23A towards hCES1A, hCES2A, hAchE and hBchE, molecular docking simulations were docked and scored by AutoDock Vina (1.1.2). As depicted in Figure 7 and Table 4, AB23A could be firmly bound to hBchE, hCES2A and hCES1A, with the predicted binding energy of −9.96, −9.67, and −8.27 kcal/mol, respectively. Meanwhile, the distances between the hydrolytic site of AB23A (the carbonyl at C-23 site) and the catalytic serine of hBchE, hCES2A and hCES1A were 3.77, 3.25, and 4.12 Å, respectively. The docking simulations results indicate that AB23A is a good substrate for hBchE and hCES2A, while can also be hydrolyzed by hCES1A. However, the distance between the hydrolytic site of AB23A (the carbonyl at C-23 site) and the catalytic serine of hAchE (11.20 Å) was much greater than the catalytic distance (<5.5 Å), while the predicted binding affinity was very low (−5.58 kcal/mol). These observations suggested that AB23A was hardly to be hydrolyzed by hAchE. The docking simulations results are consistent with the experimental results, in which hBchE and two carboxylesterases (hCES1A and hCES2A) play key roles in AB23A hydrolysis in human tissues.

**TABLE 4:** The molecular docking simulations of AB23A towards hBchE, hCES2A, hCES1A, hAchE.

Enzyme	Crystal structure	Affinity (kcal/mol)	Distance (Å)
hBchE	1p0i	−9.96	3.77
hCES2A	Homology modeling	−9.67	3.25
hCES1A	2dr0	−8.27	4.12
hAchE	4ey7	−5.58	11.20

## 4 Discussion

Mammalian esterases are the important class of serine hydrolases, which catalyze hydrolytic metabolism of numerous xenobiotics bearing ester or amide bond(s) to release a polar carboxylic acid metabolite and a hydroxyl metabolite. In humans, the turnover rates of most known esterases (such as hBchE and human carboxylesterases) are generally faster than that of cytochrome P450 enzymes (de Man et al., 2018; Hedges et al., 2019). As the results, the esterase-catalyzed hydrolytic metabolism is considered to be the first step and a predominant metabolic pathway for the agents bearing ester bond(s), such as aspirin, clopidogrel, and remdesivir (Zhang et al., 2022). In this study, the hydrolytic pathways of AB23A (a major bioactive constituent in the Chinese herb Zexie) and the key enzymes involved in AB23A hydrolysis were well-characterized for the first time. The results indicated that AB23A could be readily hydrolyzed to AB in a panel of human biological systems, including human plasma, intestinal and hepatic preparations. Meanwhile, hBchE, hSA and two human carboxylesterases (hCES1A, hCES2A) were identified as the key enzymes responsible for AB23A hydrolysis in humans. Considering that hBchE is widely distributed in human plasma and liver, while hCES1A and hCES2A are the most abundant serine hydrolases distributed in human liver and intestine, it is easily conceivable that AB23A can be rapidly hydrolyzed in humans.

Over the past decade, significant species differences in the function and inhibitor response of mammalian esterases have been well documented. Especially, the inhibitory effects of various esterase inhibitors on the hydrolysis of esterase substrates varies significantly among different species. Some specific inhibitors against human esterases do not work in tissue preparations from rodents. In these cases, this study only deciphers the contribution of each human esterase in AB23A hydrolysis by using a set of specific chemical inhibitors of some important human esterases (such as hBchE, hCES1A, and hCES2A). Although hBchE has been found playing a predominant role in AB23A hydrolysis in human plasma, hSA (the most abundant plasma protein) may also contribute this reaction to a certain extent in human plasma. It is well-known that hSA is a unique pseudo-esterase, the acyl group of hSA substrate will be covalently modify the lysines of hSA. The unique hSA-catalyzed hydrolysis generates a panel of chemically stable hSA-acyl adducts, while these adducts are very stable under physiological conditions, showing very long metabolic half-lives (Liyasova et al., 2010). Notably, it has been reported that some lysine residues of hSA (such as Lys-195 and Lys-199) are important for ligand-binding and the enzymatic activity of this key plasma protein (Muste and Gu, 2022), the high levels of acyl modified hSA in human plasma may bring undesirable effects. Thus, the influence of AB23A on the biological functions of hSA needs further investigations. Considering that the substrate preferences of several important human esterases (such as hBchE, hCES1A, and hCES2A) and hSA have been well-investigated, the medicinal chemists can design and develop novel ester prodrugs of AB that is hydrolyzed by a particular esterase rather than hSA. In the future, more efforts should be made to design a smart ester-bearing prodrug of AB that can be released by a particular esterase (rather than hSA) in a target tissue or a specific cell type.

As a potent naturally occurring FXR agonist, AB23A has been found with a variety of biological effects including the protective effects of renal ischemia-reperfusion injury and estrogen-induced cholestatic liver injury and liver regeneration (Meng et al., 2014; Jin et al., 2017; Chen et al., 2020; Luan et al., 2021). It is well-known that FXR is a crucial target to regulate bile acid metabolism, lipid and glucose homeostasis, as well as to ameliorate the inflammatory responses, non-alcoholic fatty liver disease (NAFLD) and atherosclerosis (Appelman et al., 2021; Trauner and Fuchs, 2022; Hucke et al., 2016; Clifford et al., 2021; Wu Q. et al., 2021). Increasing evidence has shown that AB23A and the Chinese herb Zexie can alleviate NAFLD in mice, but this agent can also be rapidly hydrolyzed to release AB in mice. In this study, our findings clearly demonstrate that both AB and AB23A have potent FXR agonist effects, suggesting that AB23A hydrolysis is not affect its FXR agonist effect *in vivo*. Interestingly, the FXR agonist effect of AB was slightly more potent than that of AB23A at low doses, suggesting that AB might be the key active substance of AB23A *in vivo*. Inspired by the similar FXR agonist effect of AB23A and AB, more ester derivatives of AB at C-23 hydroxyl group should be designed and synthesized, aiming to develop more efficacious agents as novel FXR agonists. In near future, the medicinal chemists can design more efficacious FXR agonists on the basis of the structure-activities of AB derivatives as FXR agonists, as well as the tissue distribution and substrate preferences of human esterases. Furthermore, the C-23 hydroxyl group of alisol B can also be esterified by a variety of carboxylic acids with anti-inflammatory and lipid-lowering activities (such as ferulic acid, caffeic acid, chlorogenic acid) to design a panel of novel ester-bearing prodrugs with dual biological functions (such as lipid-lowering and FXR agonist effect), to develop novel prodrugs for treating NAFLD or obesity-related metabolic disorders.

## 5 Conclusion

In summary, the study revealed the metabolic organs and the key enzymes responsible for AB23A hydrolysis in humans, as well as tested the FXR agonist effects of both AB23A and its hydrolytic metabolite AB. Our findings clearly indicated that AB23A could be hydrolyzed to release AB in human plasma and a panel of tissue preparations (HLMs and HIMs). Reaction phenotyping and chemical inhibition assays suggested that hBchE played a predominant role in AB23A hydrolysis in human plasma and hSA contributed to this reaction a lesser extent, while carboxylesterases (including hCES1A and hCES2A) played key roles in AB23A hydrolysis in human intestine and liver preparations. FXR agonist activity tests revealed that AB showed similar FXR agonist effect to AB23A, suggesting that AB23A hydrolysis did not affect its FXR agonist effect. Collectively, the hydrolytic pathways of AB23A in human biological systems are well-characterized, which will be very helpful for the pharmacologists to deeply understand the metabolic rates of AB23A in humans, as well as useful for the medicinal chemists to develop novel ester prodrugs of AB for specific purposes.

## Data Availability

The datasets presented in this study can be found in online repositories. The names of the repository/repositories and accession number(s) can be found in the article/Supplementary Material.

## Abbreviations

AB23A, Alisol B 23-acetate; AB, alisol B; BNPP, bis(p-nitrophenyl) phosphate; CA, carbonic anhydrase; CYPs, cytochrome P450 enzymes; EDTA, ethylenediaminetetraacetic acid; FXR, farnesoid X receptor; GA, galanthamine; hAchE, human acetylcholinesterase; hBchE, human butyrylcholinesterase; hCES1A, human carboxylesterase 1A; hCES2A, human carboxylesterase 2A; hCTSA, human cathepsin A; hIAH1, human isoamyl acetate-hydrolyzing esterase 1; HIMs, human intestine microsomes; HKMs, human kidney microsomes; HLMs, human liver microsomes; hMAGL, human monoacylglycerol lipase; hNotum, human Notum; hPL, human pancreatin lipase; hSA, human serum albumin; LPA, loperamide; MLMs, mouse liver microsomes; NAFLD, non-alcoholic fatty liver disease; PLMs, pig liver microsomes; PBS, phosphate-buffered saline; RLMs, rat liver microsomes.
