# Supplementary material for: The roles of serine hydrolases and serum albumin in alisol B 23-acetate hydrolysis in humans
Source: Front Pharmacol. 2023 Apr 6;14:1160665. doi: 10.3389/fphar.2023.1160665 (PMC10117764; doi:10.3389/fphar.2023.1160665)
Supplement: Supplementary file 1 [file DataSheet1.docx]

Supplementary Material

**The roles of serine hydrolases and serum albumin in alisol B 23-acetate hydrolysis in humans**

Tiantian Zhang^1,2,†^, Feng Zhang^2,3,†^, Yani Zhang^2^, Hongxin Li^2^, Guanghao Zhu^2^, Taotao Weng^3^, Cheng Huang^4^, Ping Wang^2^, Yuqi He^1^, Jing Hu^3*^ and Guangbo Ge^1,2,*^

^1^School of Pharmacy, Zunyi Medical University, Zunyi, Guizhou 563000, China

^2^Shanghai Frontiers Science Center of TCM Chemical Biology, Institute of Interdisciplinary Integrative Medicine Research, Shanghai University of Traditional Chinese Medicine, Shanghai 201203, China.

^3^Department of Nephrology, The Seventh People's Hospital of Shanghai University of Traditional Chinese Medicine, Shanghai 200137, China.

^4^School of Chinese Medicine, Shanghai University of Traditional Chinese Medicine, Shanghai 201203, China.

*** Correspondence:**

Corresponding Author

E-mail: geguangbo@shutcm.edu.cn (G.B. Ge), hujing@shutcm.edu.cn (J. Hu)

**^†^** These authors contributed equally to this work and share first authorship

# Supplementary experimental

# 1 Expression and purification of human hydrolases

# 1.1 Expression and purification of hCES1A

# The cDNA of human CES1A, with an amino acid sequence of 18-563, truncated 4 amino acids at the C-terminal and fused with 10xHis-tag, was cloned into the pTT5 vector after codon optimization (plasmid synthesis company: GENEWIZ). HEK293-F cells were cultured in OPM-293 CDO5 Medium (Shanghai OPM Biosciences, Ltd) at 5% CO2, 37 ºC and 130 rpm. When the cell density reached 2.0×10^6^ cells/mL, the human CES1A plasmid was added to start transfection. After 72 hours, the culture medium was collected by centrifugation for 10 min at 16000 g. The conditioned medium is purified by a metal affinity chromatography purification column to obtain purified human recombinant expression CES1A. The enzymatic kinetics of recombinant hCES1A were plotted by using NLMe as a probe substrate (Morton et al., 2000).

# 1.2 Expression and purification of hCES2A

# The cDNA of human CES2A, amino acid sequence 27-559, fused with 10xHis-tag, was cloned into pTT5 vector after codon optimization (plasmid synthesis company: GENEWIZ). HEK293-F cells were cultured in OPM-293 CDO5 Medium (Shanghai OPM Biosciences, Ltd) at 5% CO_2_, 37 ºC and 130 rpm. When the cell density reached 2.0×10^6^ cells/mL, the human CES2A plasmid was added to start transfection. After 96 hours, the culture medium was collected by centrifugation for 10 min at 16000 g. The conditioned medium is purified by a metal affinity chromatography purification column to obtain purified hCES2A (Lamego et al; 2013).

# 1.3 Expression and purification of hPL

# The human pancreatic cDNA was cloned into the pTT5 vector with the N-terminal mouse IgG κ chain signal peptide and C-terminal His10-tag. Freestyle 293-F cells (Invitrogen) were cultured in SMM 293T-II medium (Sino Biological Inc.) at 37 ºC, 130 rpm under 5% CO_2_. The pTT5 containing human pancreatic lipase cDNA plasmid was pre-mixed with PEI MAX 40K (Polysciences) for 30 min before transfection. Transfection was started by adding plasmid-PEI MAX 40K mixture when the cell density reached 2.0×10^6^ cells/mL. After 72 h, the transfected cells were centrifuged at 1000 rpm for 10 min and the conditioned medium was collected. The conditioned medium was loaded on the Ni Sepharose excel resin (GE Healthcare). The resin was washed with wash buffer 1 containing 25 mM Tris-HCl, pH 7.4, 150 mM NaCl, 1 mM CaCl_2_, 2 mM imidazole. The protein was eluted with elute buffer 1 containing 25 mM Tris-HCl, pH 7.4, 150 mM NaCl, 1 mM CaCl_2_, 200 mM imidazole. The eluted protein was diluted 10 times with a buffer (25 mM Tris-HCl, pH 8.0, 150 mM NaCl, 1 mM CaCl_2_) and incubated with Ni-NTA agarose (Qiagen) for further purification. The agarose was washed with wash buffer 2 containing 25 mM Tris-HCl, pH 8.0, 150 mM NaCl, 1 mM CaCl_2_, 20 mM imidazole. The protein was then eluted with elute buffer 2 containing 25 mM Tris-HCl, pH 8.0, 150 mM NaCl, 1 mM CaCl_2_, 300 mM imidazole. The fractions with hPL were concentrated using a 10 kDa molecular weight cut-off (MWCO) concentrator (Millipore). The concentrated sample was purified on a Superdex 200 10/300 GL column (GE Healthcare) equilibrated in protein storage buffer (25 mM Tris-HCl, pH 7.4, 150 mM NaCl, 1 mM CaCl_2_).

# 1.4 Incubation conditions of AB23A and its hydrolytic metabolite

# The incubation conditions were as follows: the classic incubation system included 100 mM potassium phosphate buffer (PBS, pH 7.4), enzyme sources, and AB23A. In all incubations, PBS and enzyme sources were pre-incubation at 37 ºC for 3 min and the reaction was started by adding AB23A (the organic solvent content at most 1%). After reacting 1 h, ice-cold acetonitrile was added to terminate the reaction and precipitate proteins. The samples were centrifuged for 30 min at 20000 × g, 4 ºC, and the supernatants were taken for further LC-UV analysis.

# 1.5 The analysis conditions of the modification sites of hSA by AB23A

# The samples were analyzed by a Q Exactive™ HF-X mass spectrometer (Thermo Scientific). About 2 µL tryptic peptides were injected into nano-LC system (EASY-nLC 1200, Thermo Fisher Scientific, USA) (Hu et al; 2021), separated through a self-packed analytical C18 column (20 µm × 360 µm × 200 mm, 3 μm) with a 65-min linear gradient at a flow-rate of 300 nL/min. The mobile phase was composed of 0.1% (*v/v*) formic acid in water (solvent A) and 80% acetonitrile-20% water (solvent B). The gradient elution programs were as follows: 0-1 min, 1%-6% B, 1-47 min, 6%-25% B, 47-54 min, 25%-37% B, 54-56 min, 37%-95% B, 56-65 min, 95% B. The MS data were collected by data-dependent mode on the Hybrid Quadrupole-Orbitrap mass spectrometer (Q Exactive TM HF-X, Thermo Fisher Scientific, USA) with a full scan from *m/z* 300-1800 and resolution of 60000 (AGC target 3e^6^, maximum IT 50 ms). The compounds were fragmented in the HCD (Higher Energy Collision Dissociation) mode with a resolution of 15000 at a 28% normalized collision energy (AGC target 1e^5^, maximum IT 30 ms).

## Supplementary Figures


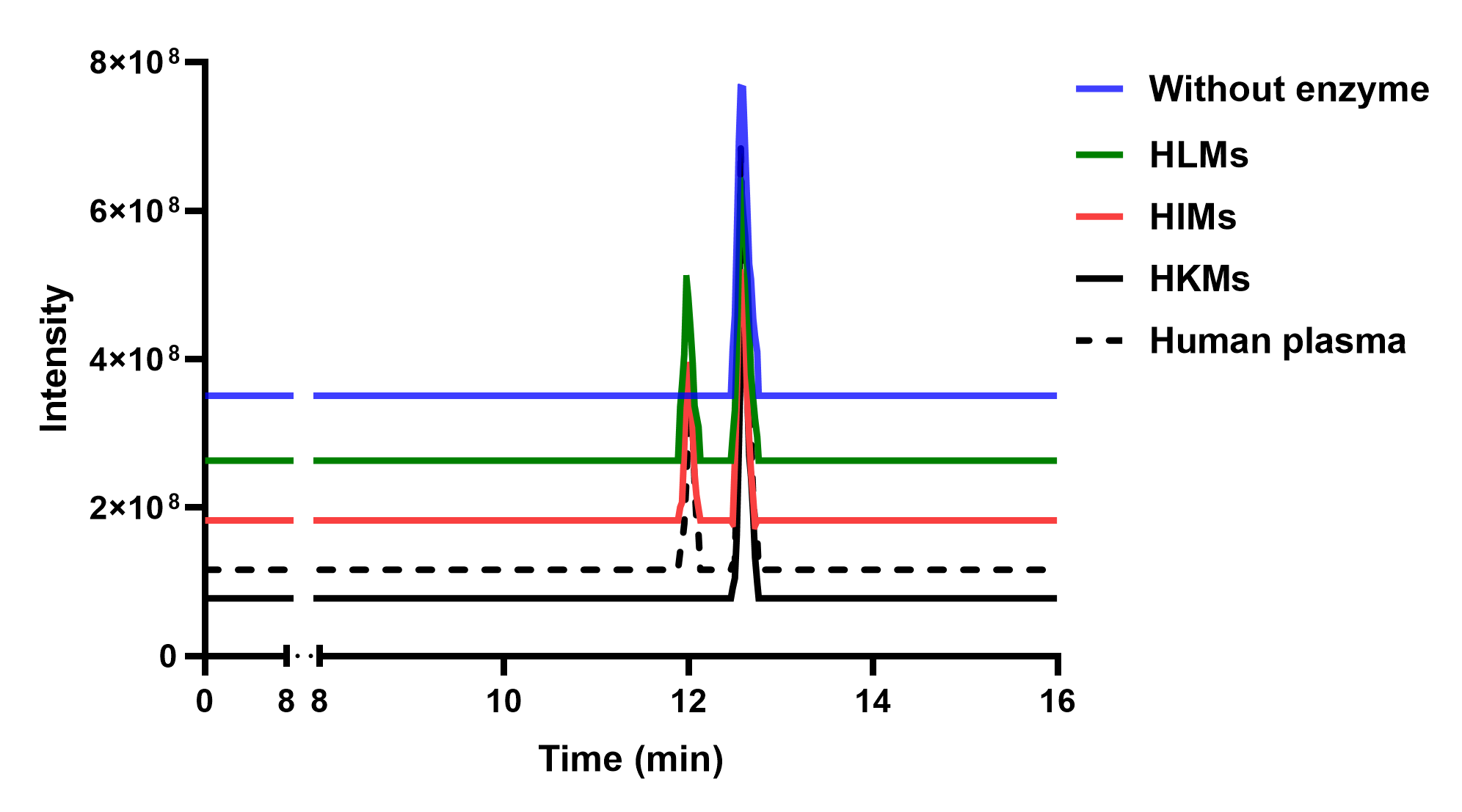


**Supplementary Figure 1**. Extracted ion chromatograph of AB23A co-incubation with HLMs, HIMs, HKMs, and human plasma (25% dilution).

***
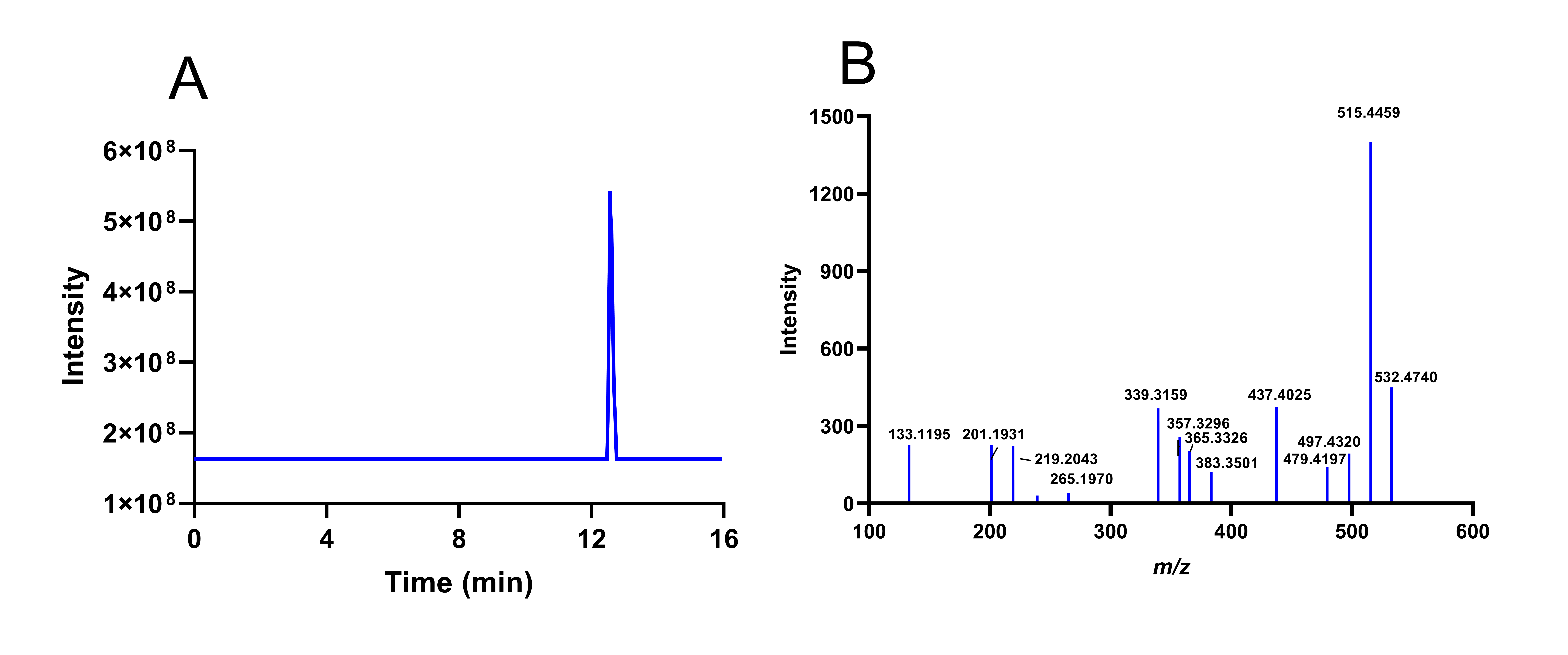
***

**Supplementary Figure 2**. Extracted ion chromatograph of AB23A **(A)**. MS/MS spectrum of AB23A **(B**).


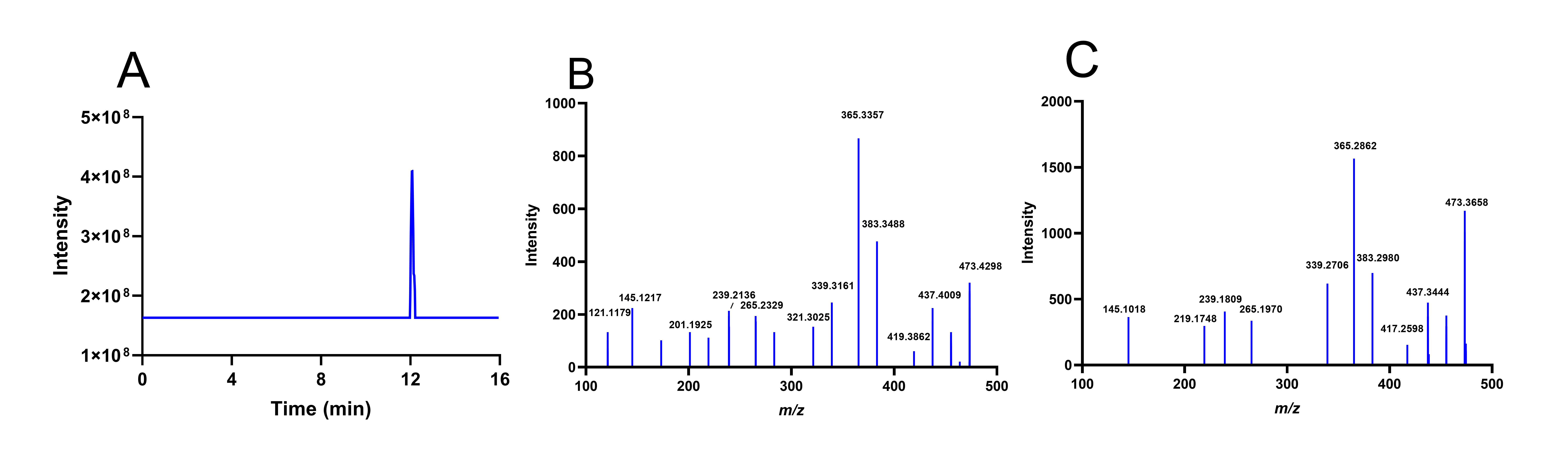


**Supplementary Figure 3**. Extracted ion chromatograph of AB **(A)**. MS/MS spectrum of AB produced in HLMs **(B)**. MS/MS spectrum of AB standard **(C)**.

**
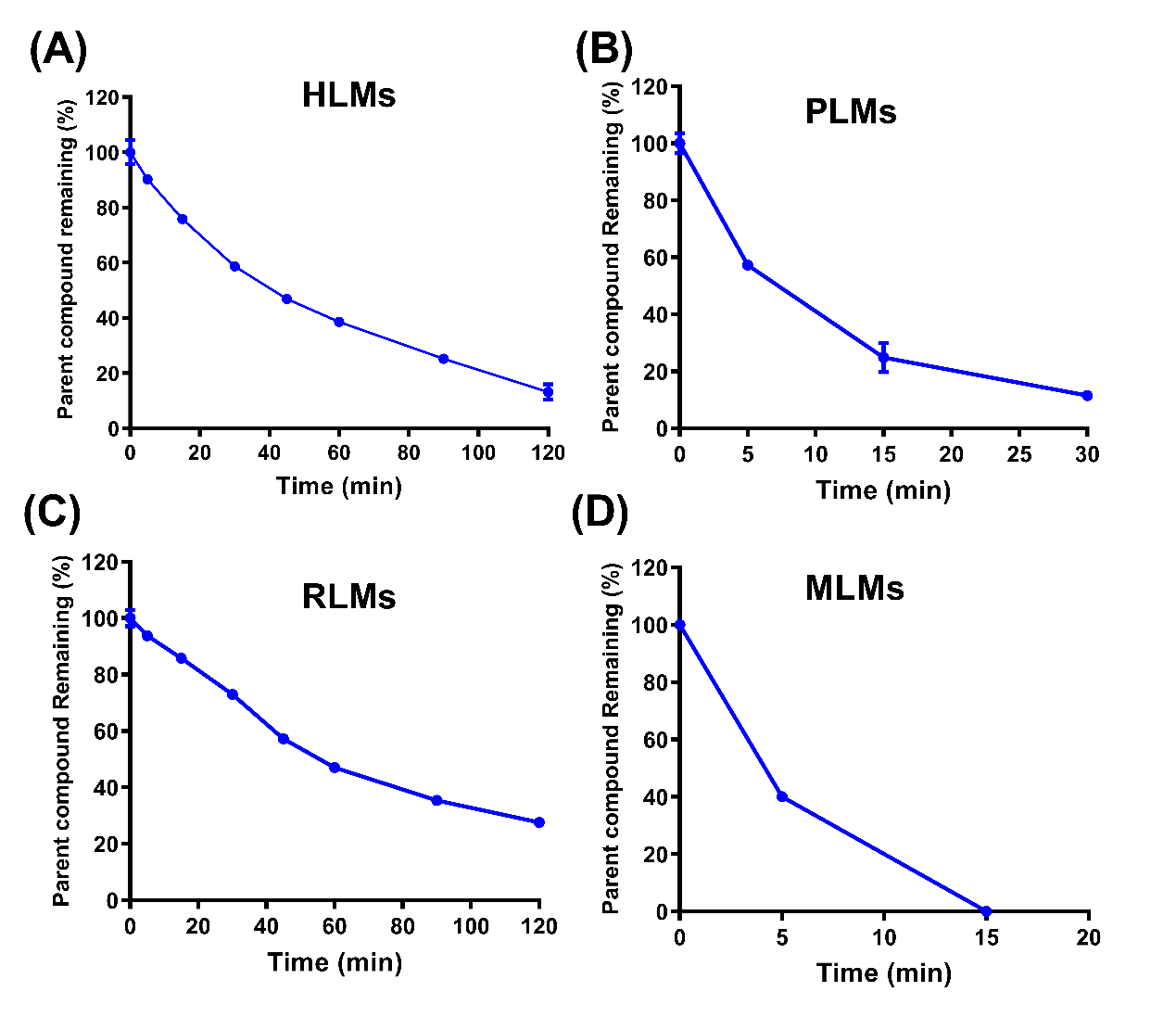
**

**Supplementary Figure 4** The metabolic half-lives of AB23A in liver microsomes from different species including HLMs **(A)**, PLMs **(B)**, RLMs **(C)** and MLMs **(D)**. All assays were conducted in triplicate, and the data were expressed as mean ± SD.

**
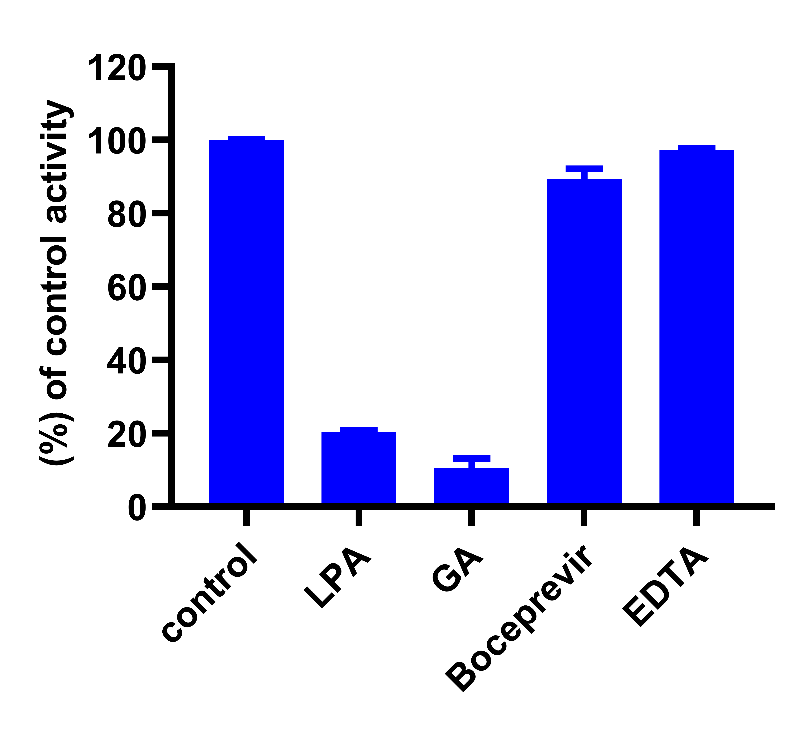
**

**Supplementary Figure 5**. Chemical inhibition assays of AB23A hydrolysis in human plasma (25% dilution).

**
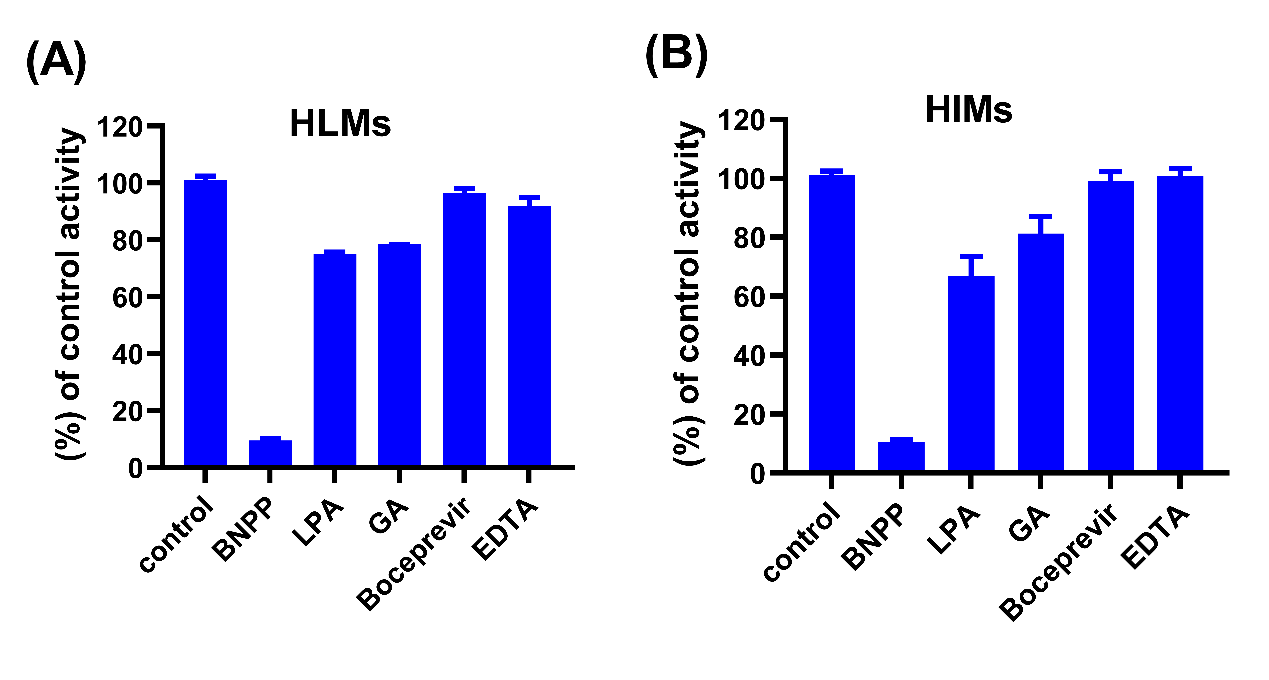
**

**Supplementary Figure 6**. Chemical inhibition assays of AB23A hydrolysis in HLMs **(A)**, HIMs **(B)**.


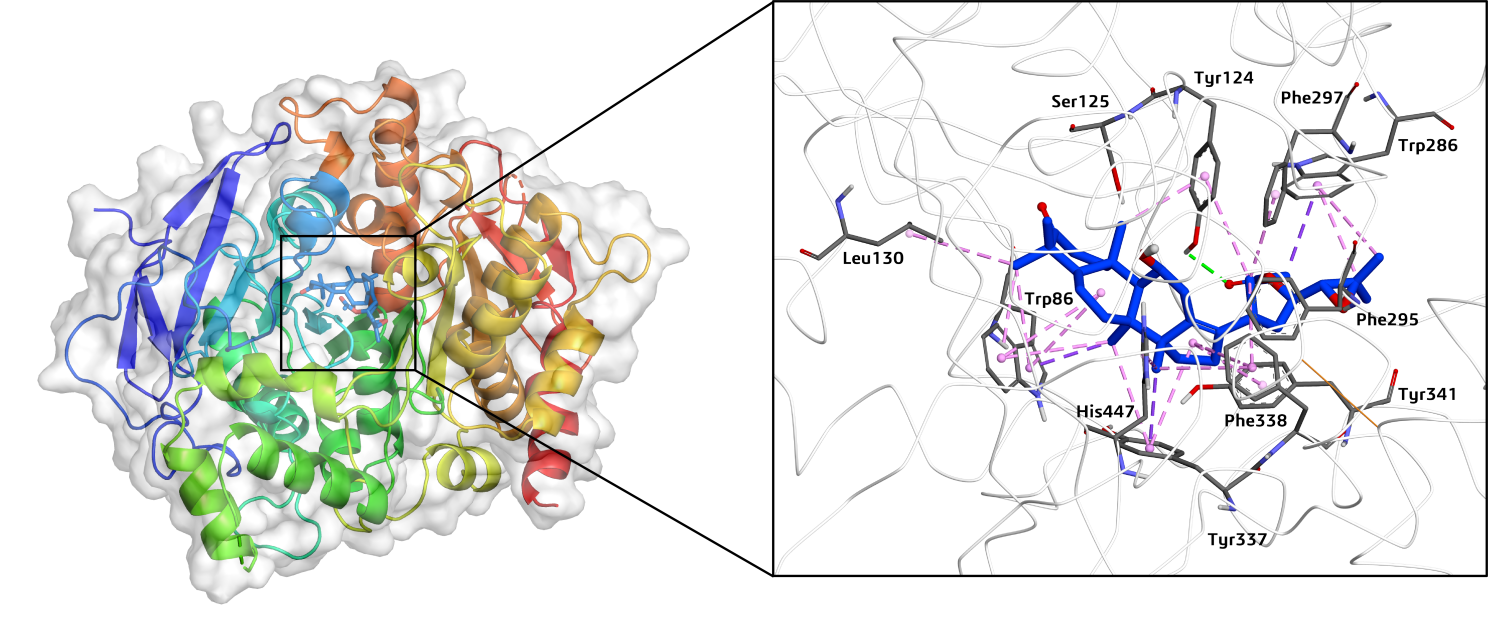


**Supplementary Figure 7.** Overall binding modes and receptor-ligand interaction analysis of AB23A (blue) in hAchE.


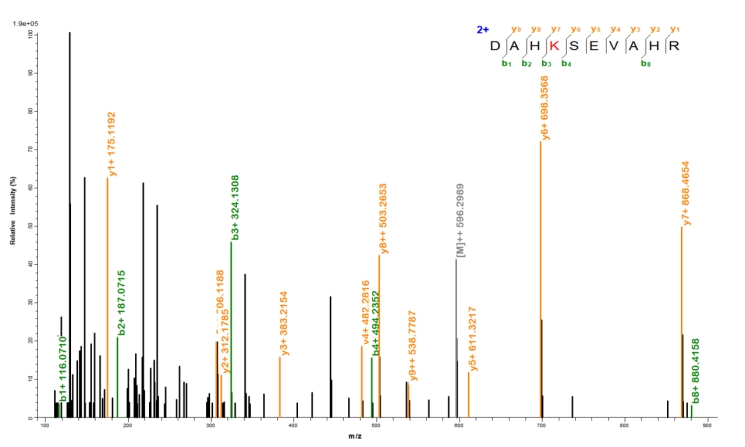

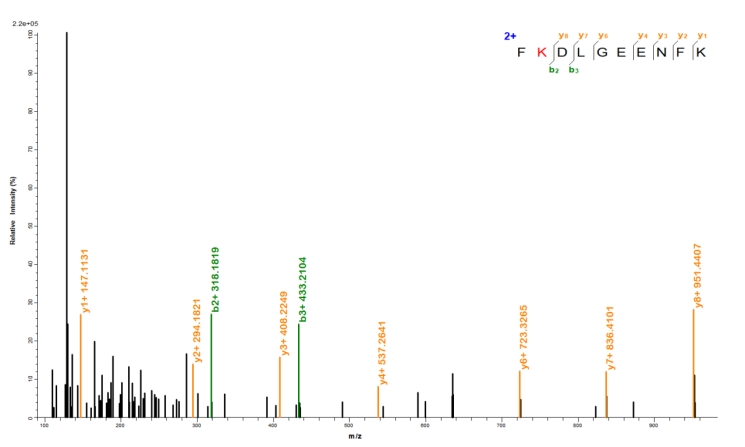

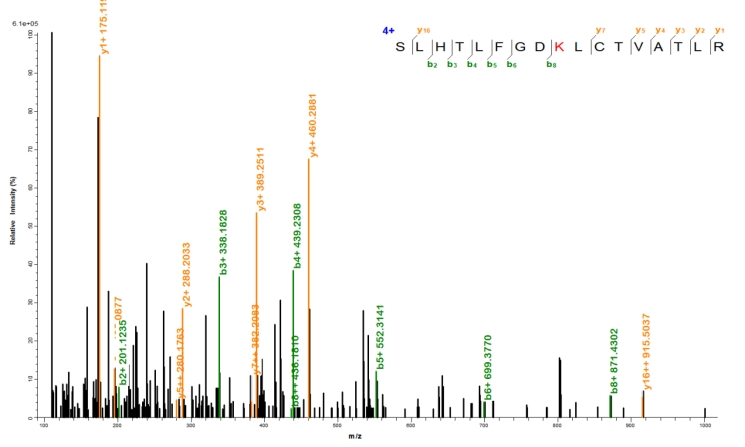

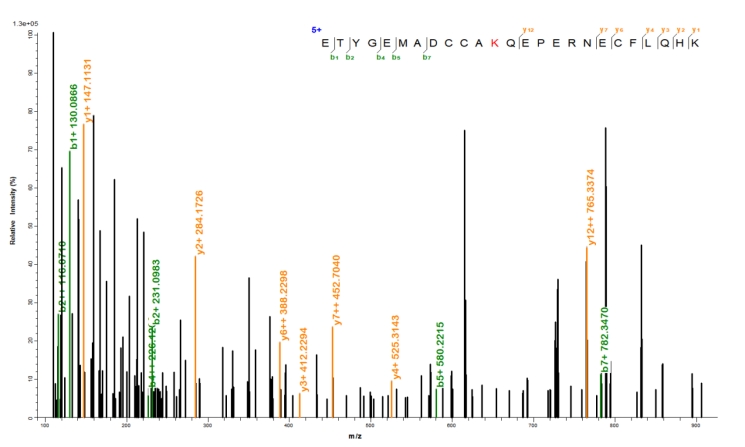

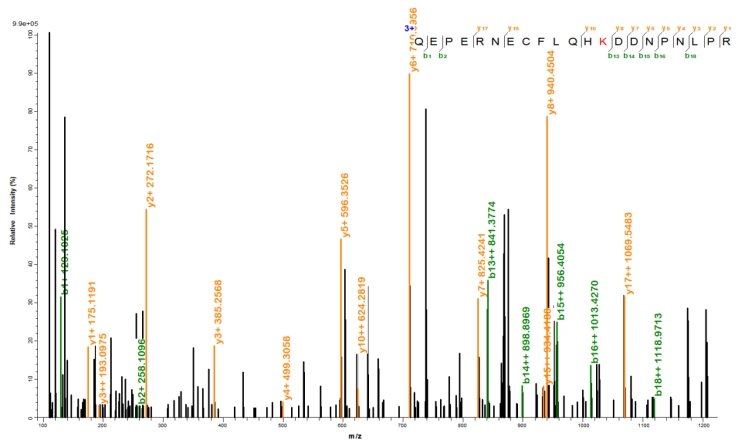

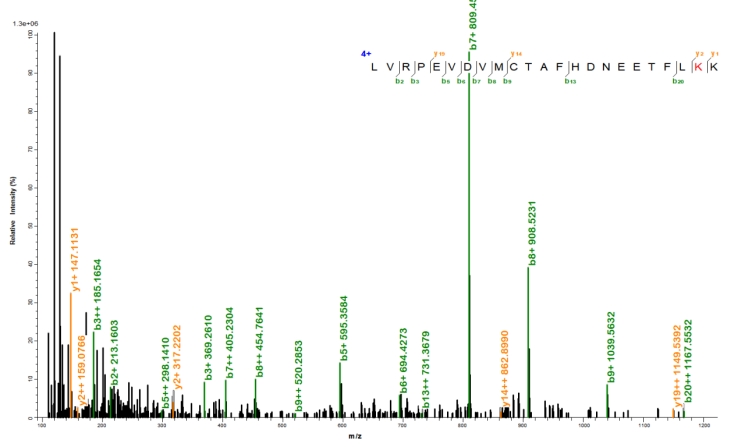

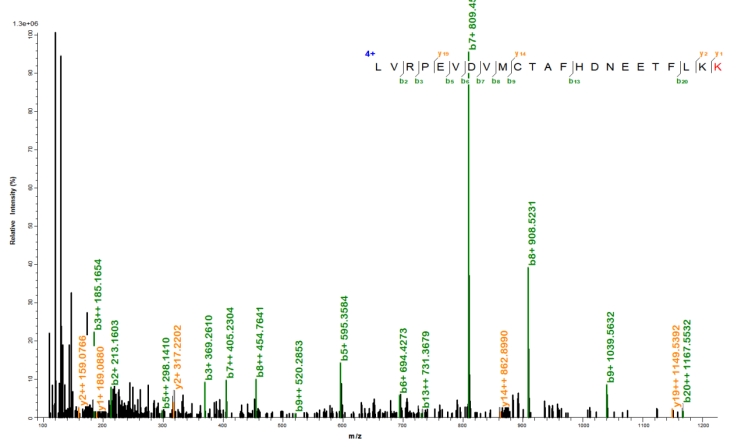

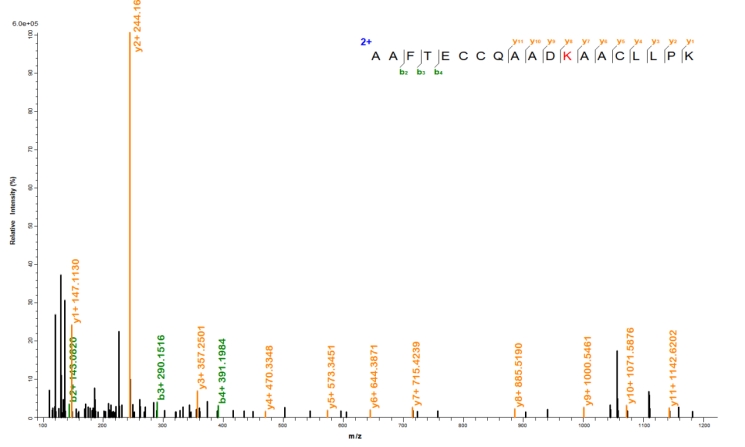

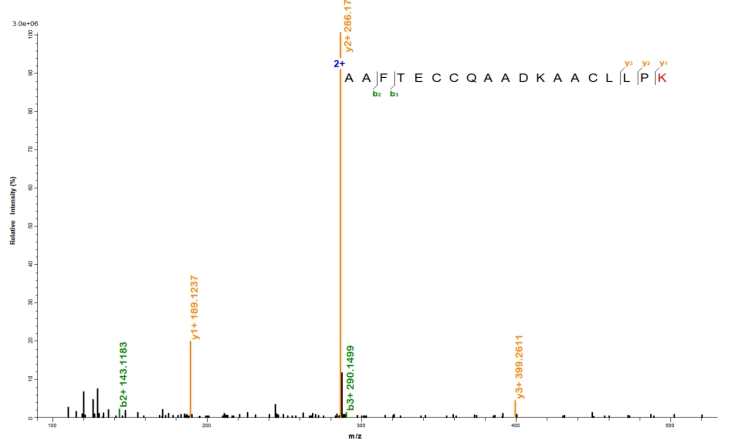

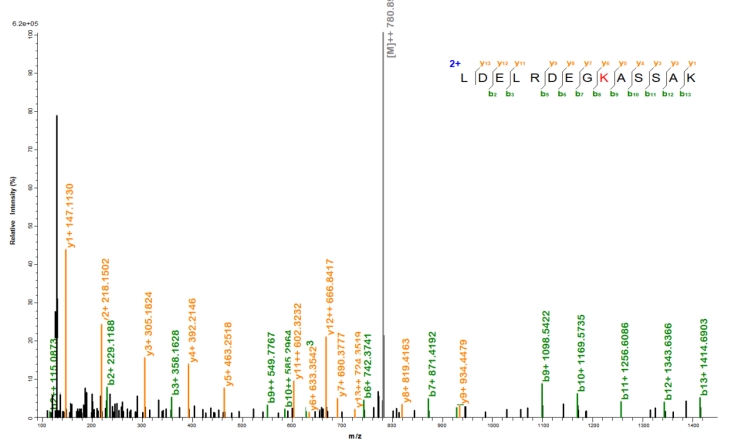

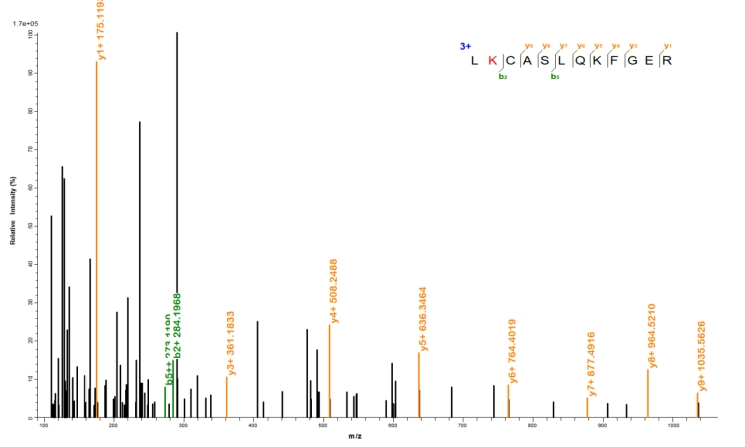

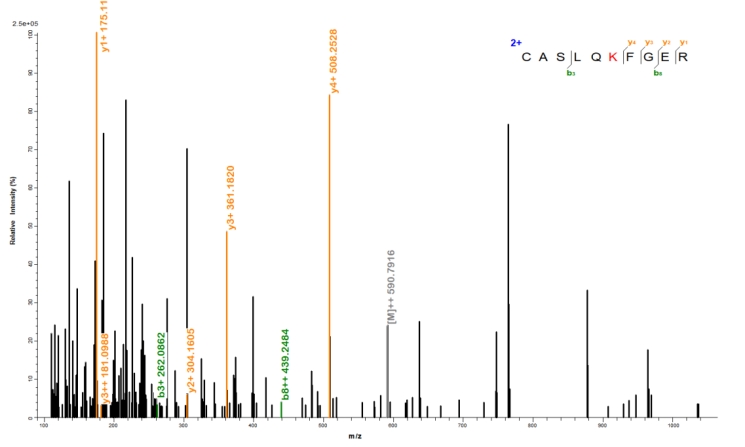

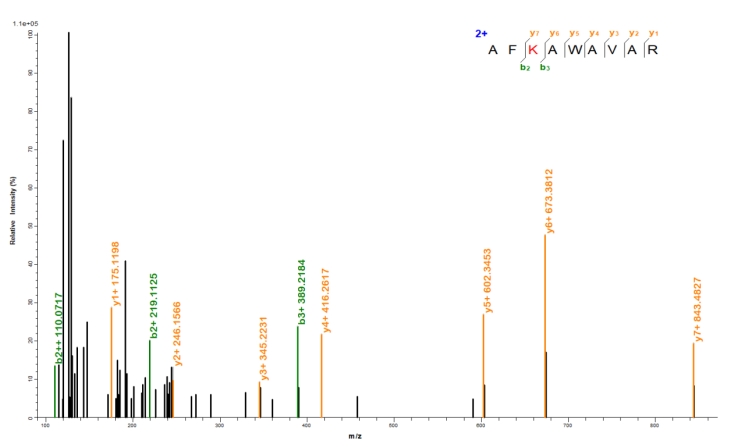

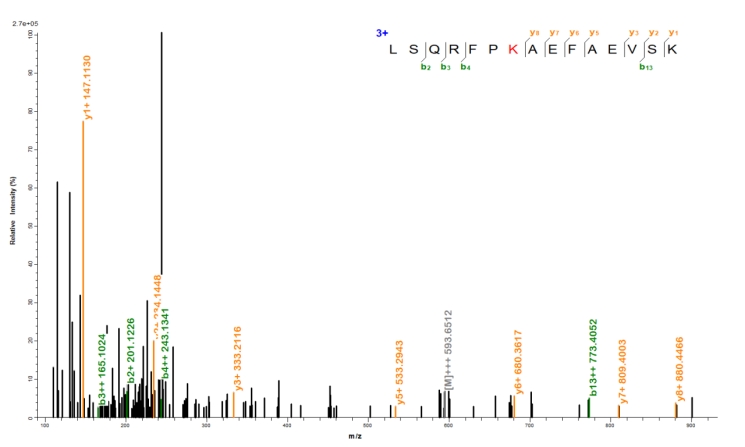

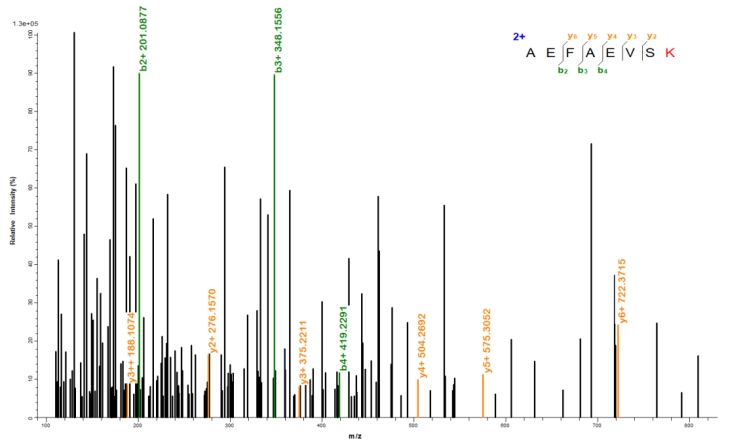

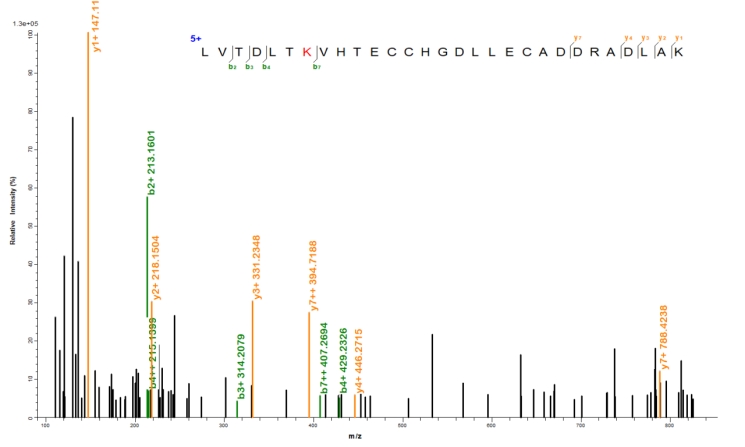

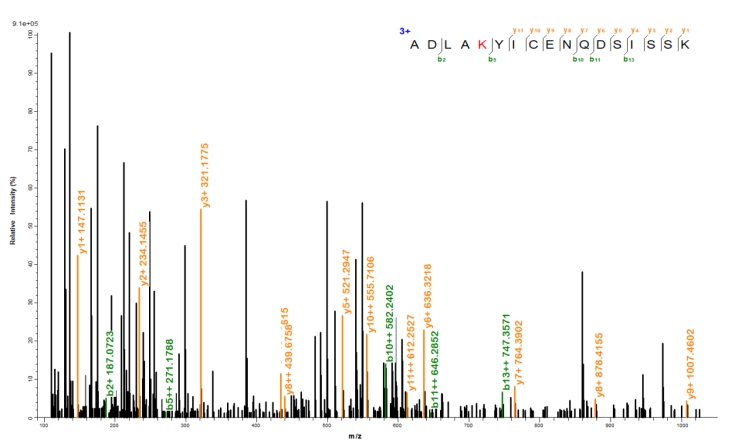

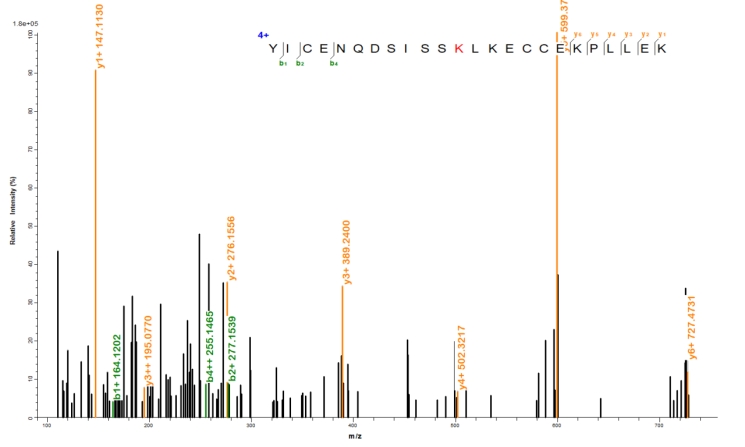

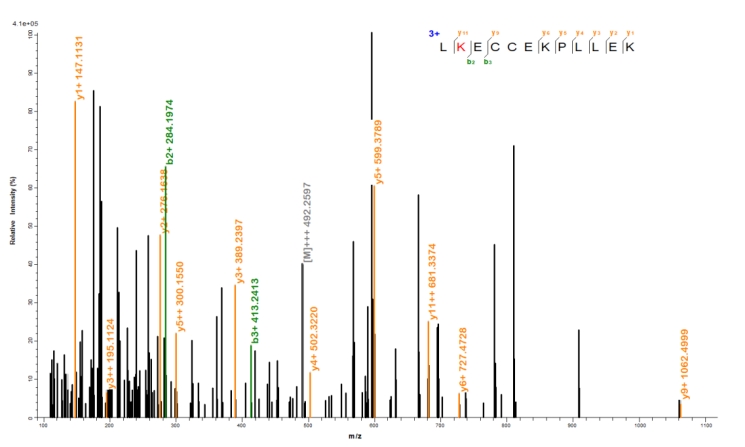

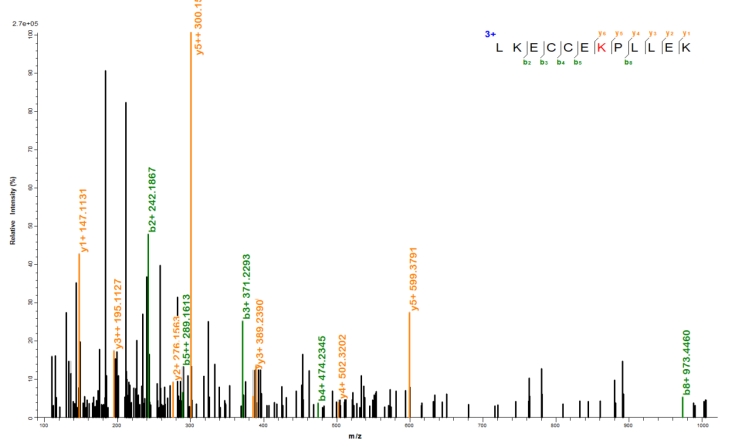

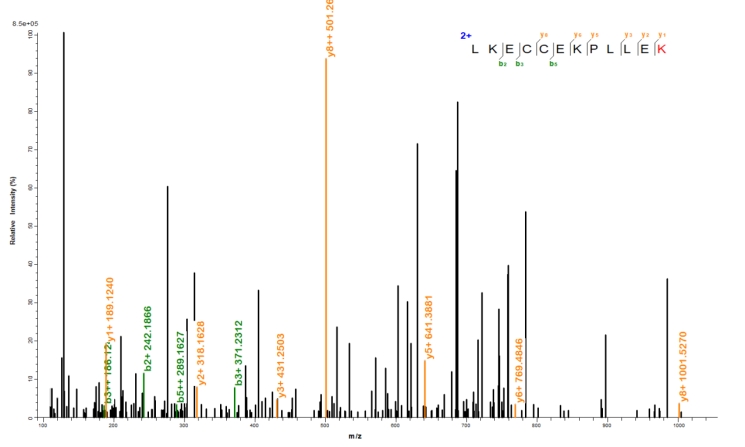

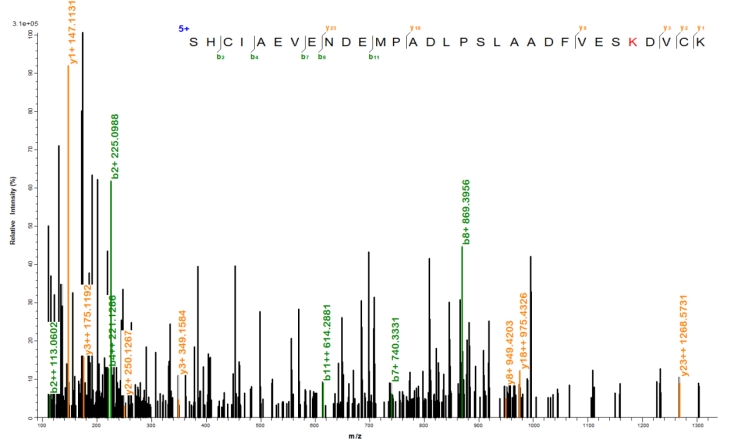

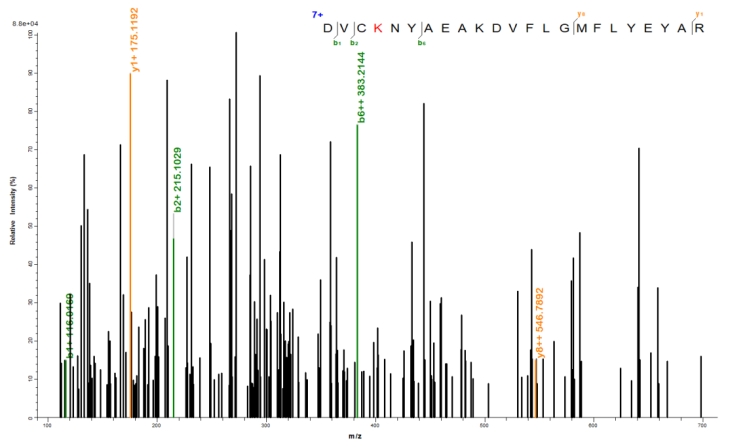

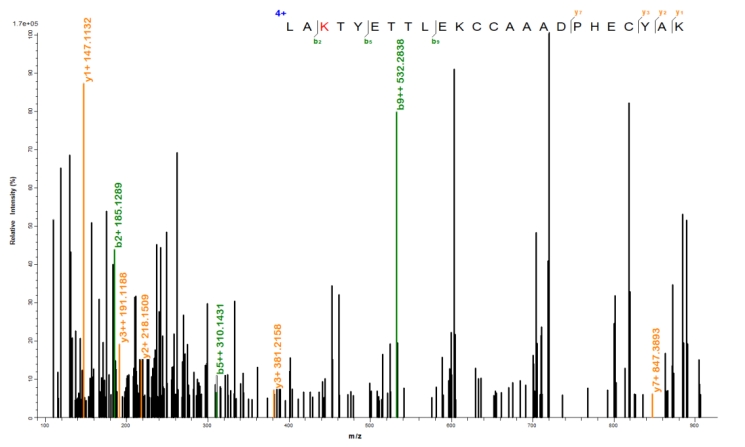

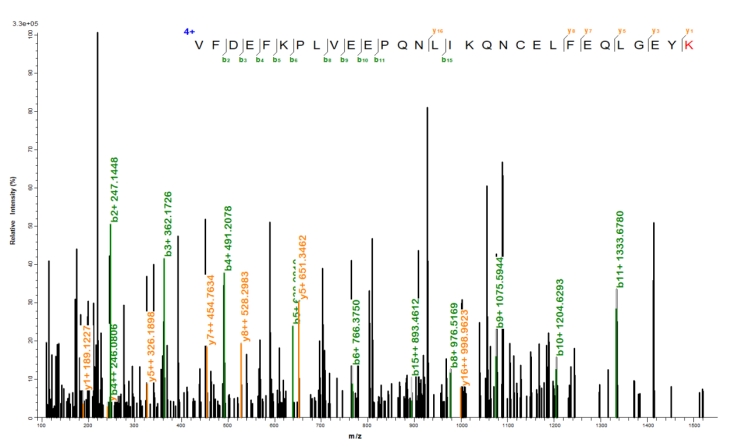

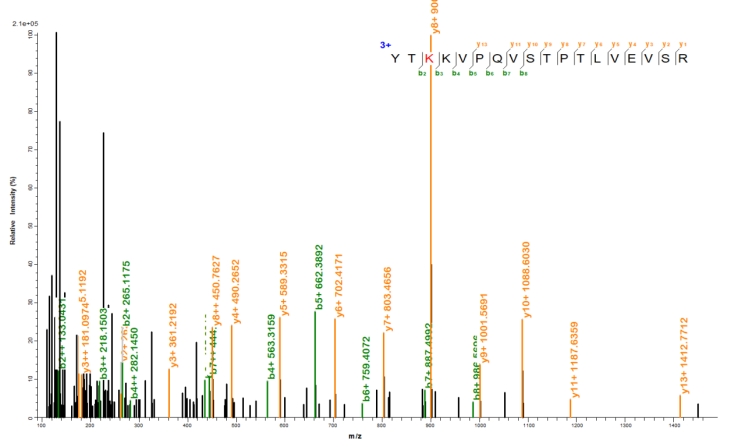

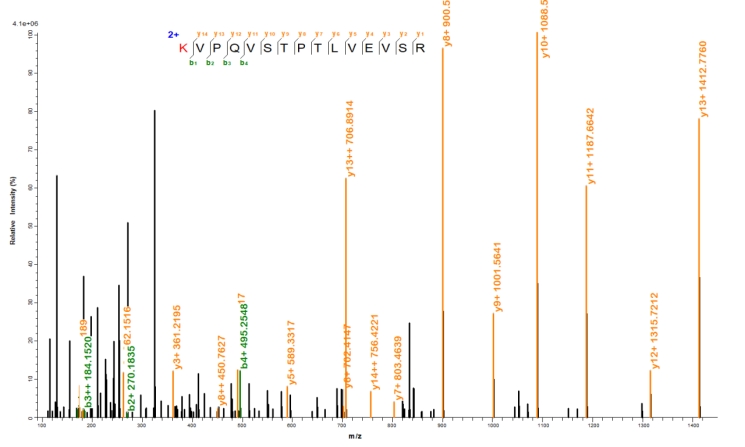

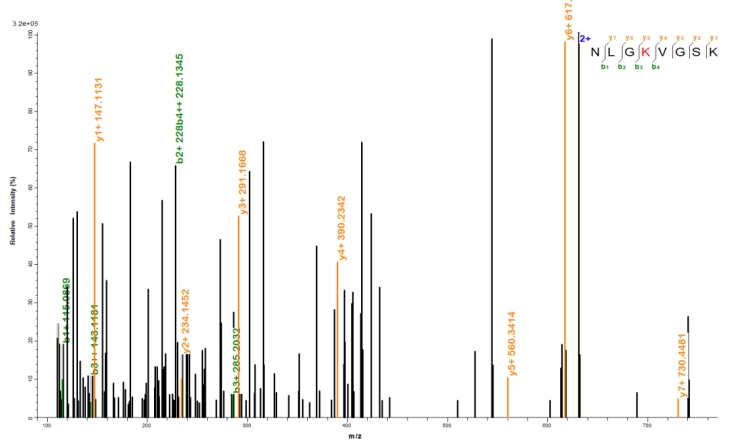

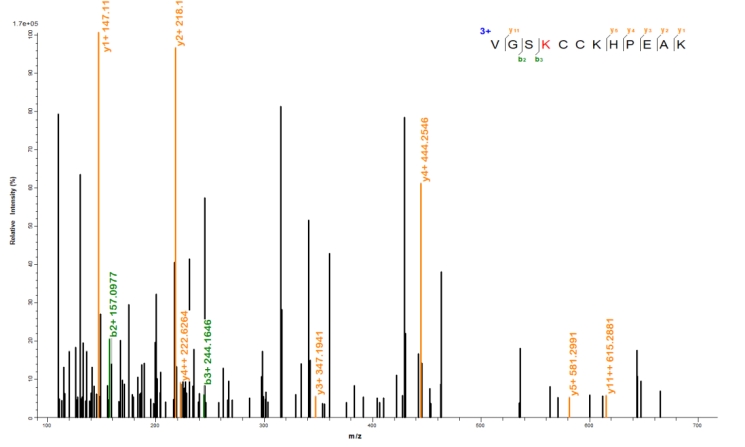

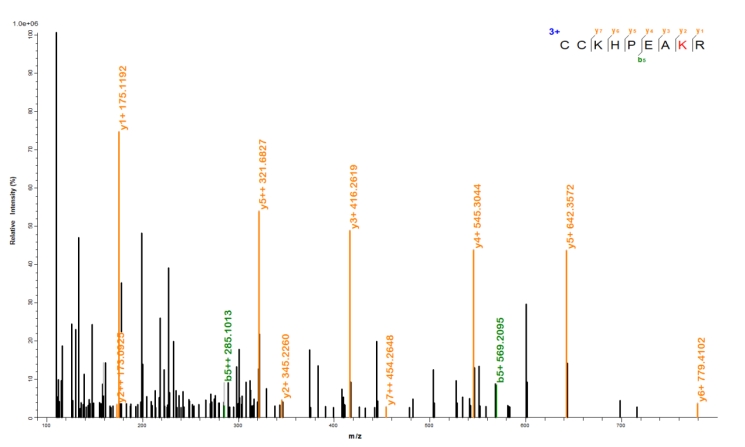

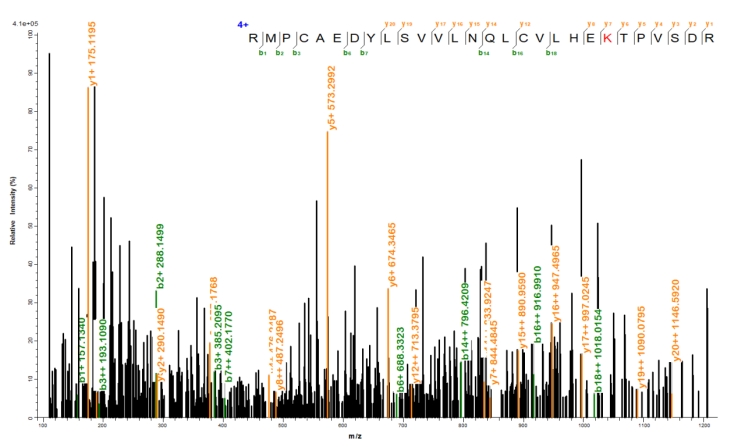

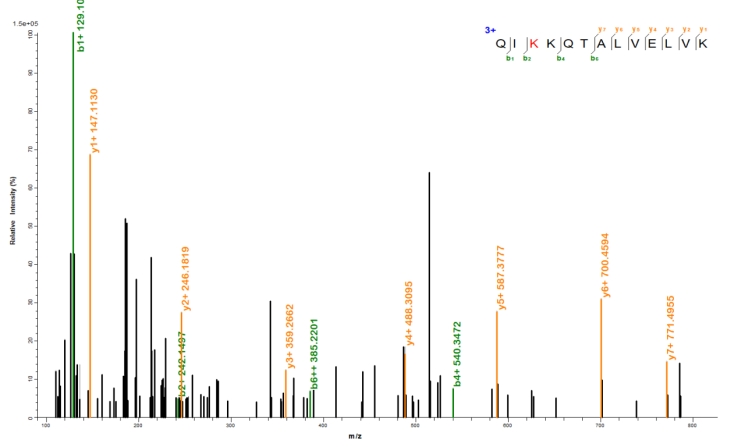

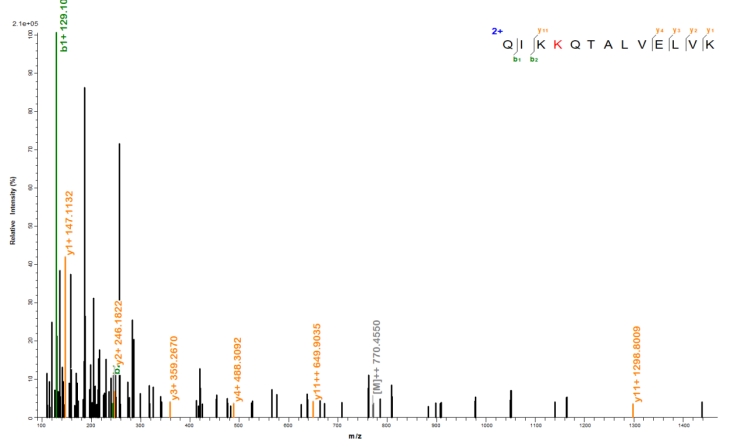

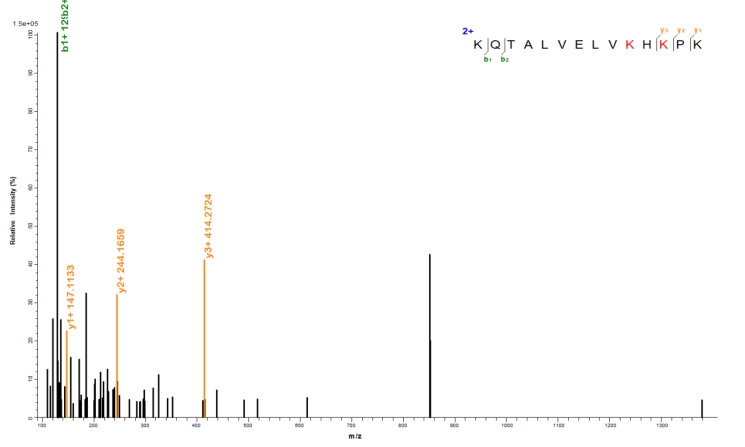

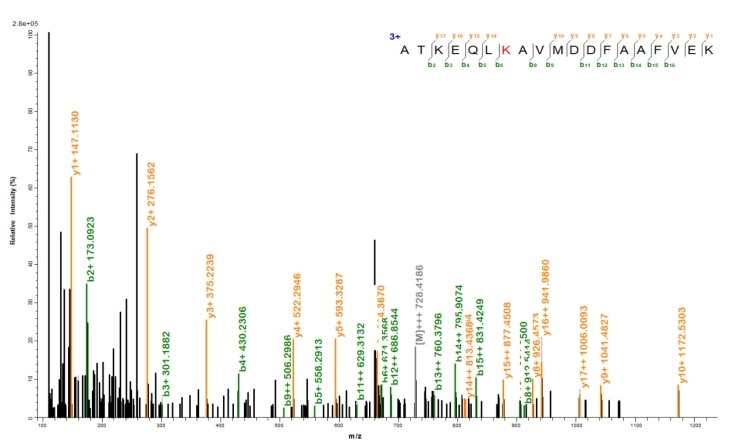

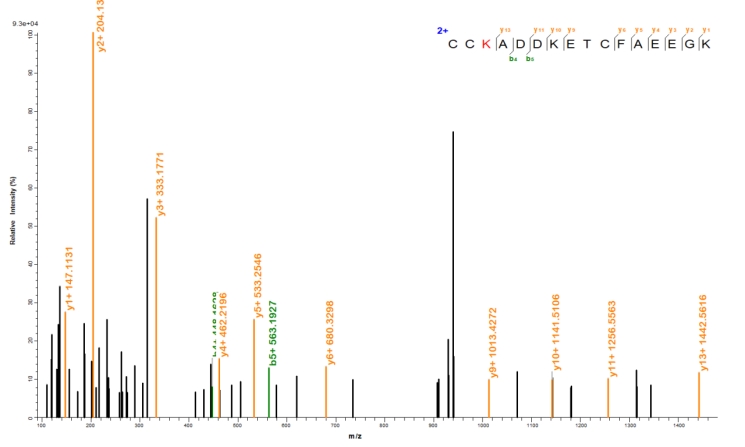

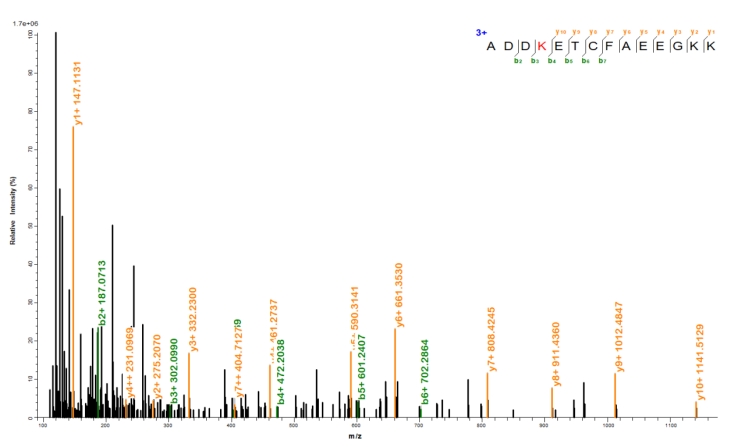

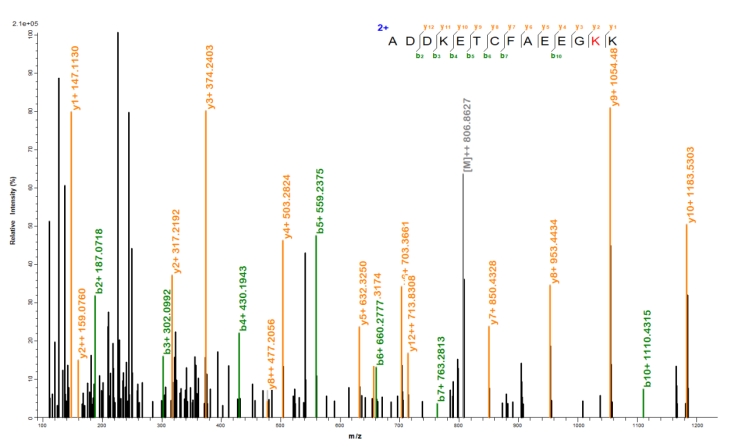

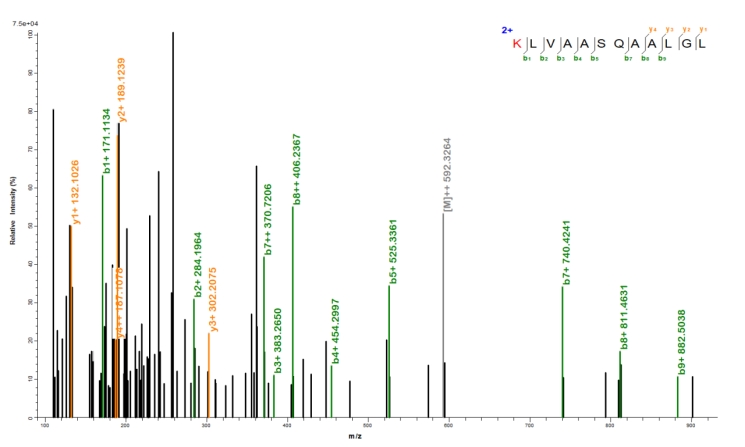


**Supplementary Figure 8**. MS/MS spectra of 39 modified peptides of human serum albumin.

**Supplementary Table 1** Hydrolytic half-lives of AB23A in liver microsomes from different animal species.

| **Enzyme sources** | **enzymes concentration**  **（mg/mL)** | **T_1/2_ (min)** |
| --- | --- | --- |
| HLMs | 0.10 | 41.00 ± 0.01 |
| PLMs | 0.10 | 9.50 ± 0.05 |
| RLMs | 0.10 | 55.63 ± 0.11 |
| MLMs | 0.10 | 2.30 ± 0.01 |

**Supplementary Table 2.** The lysine residuals of human albumin that can be acetylated by alisol B 23-acetate following 2 h incubation with human albumin.

| **No.** | **Sequence position** | **Peptide sequence** | **Modifications sites** | **Acetylated**  **lysine** | **Mass of unlabeled**  **peptide, m/z** | **Mass of labeled peptide**  **(mass shift 42), m/z** | **charge** |
| --- | --- | --- | --- | --- | --- | --- | --- |
| 1 | 25-34 | DAHK*SEVAHR | K4(Acetyl) | 28 | 575.29694 | 596.29694 | 2 |
| 2 | 35-44 | FK*DLGEENFK | K2(Acetyl) | 36 | 613.81189 | 634.81189 | 2 |
| 3 | 89-105 | SLHTLFGDK*LCTVATLR | K9(Acetyl) | 97 | 469.51572 | 480.01572 | 4 |
| 4 | 106-130 | ETYGEMADCCAK*QEPERNECFLQHK | K12(Acetyl) | 117 | 607.2662 | 615.6662 | 5 |
| 5 | 118-138 | QEPERNECFLQHK*DDNPNLPR | K13(Acetyl) | 130 | 860.4021 | 874.4021 | 3 |
| 6 | 139-161 | LVRPEVDVMCTAFHDNEETFLK*K | K22(Acetyl) | 160 | 695.58911 | 706.08911 | 4 |
| 7 | 139-161 | LVRPEVDVMCTAFHDNEETFLKK* | K23(Acetyl) | 161 | 695.58911 | 706.08911 | 4 |
| 8 | 187-205 | AAFTECCQAADK*AACLLPK | K12(Acetyl), | 198 | 1034.4917 | 1055.4917 | 2 |
| 9 | 187-205 | AAFTECCQAADKAACLLPK* | K19(Acetyl) | 205 | 1034.49536 | 1055.49536 | 2 |
| 10 | 206-219 | LDELRDEGK*ASSAK | K9(Acetyl) | 214 | 759.89825 | 780.89825 | 2 |
| 11 | 222-233 | LK*CASLQKFGER | K2(Acetyl) | 223 | 479.59921 | 493.59921 | 3 |
| 12 | 224-233 | CASLQK*FGER | K6(Acetyl) | 229 | 569.79126 | 590.79126 | 2 |
| 13 | 234-242 | AFK*AWAVAR | K3(Acetyl) | 236 | 510.29938 | 531.29938 | 2 |
| 14 | 243-257 | LSQRFPK*AEFAEVSK | K7(Acetyl) | 249 | 579.65454 | 593.65454 | 3 |
| 15 | 250-257 | AEFAEVSK* | K8(Acetyl) | 257 | 440.73047 | 461.73047 | 2 |
| 16 | 258-286 | LVTDLTK*VHTECCHGDLLECADDRADLAK | K7(Acetyl) | 264 | 660.52059 | 668.92059 | 5 |
| 17 | 282-298 | ADLAK*YICENQDSISSK | K5(Acetyl) | 286 | 647.97626 | 661.97626 | 3 |
| 18 | 287-310 | YICENQDSISSK*LKECCEKPLLEK | K12(Acetyl) | 298 | 715.10388 | 725.60388 | 4 |
| 19 | 299-310 | LK*ECCEKPLLEK | K2 (Acetyl) | 300 | 478.25647 | 492.25647 | 3 |
| 20 | 299-310 | LKECCEK*PLLEK | K7(Acetyl) | 305 | 497.26929 | 511.26929 | 3 |
| 21 | 299-310 | LKECCEKPLLEK* | K12(Acetyl) | 310 | 745.39642 | 766.39642 | 2 |
| 22 | 311-341 | SHCIAEVENDEMPADLPSLAADFVESK*DVCK | K27(Acetyl) | 337 | 699.32021 | 707.72021 | 5 |
| 23 | 338-360 | DVCK*NYAEAKDVFLGMFLYEYAR | K4(Acetyl) | 341 | 401.20117 | 407.20117 | 7 |
| 24 | 373-396 | LAK*TYETTLEKCCAAADPHECYAK | K3(Acetyl) | 375 | 708.32324 | 718.82324 | 4 |
| 25 | 397-426 | VFDEFKPLVEEPQNLIKQNCELFEQLGEYK* | K30(Acetyl) | 426 | 907.46185 | 917.96185 | 4 |
| 26 | 435-452 | YTK*KVPQVSTPTLVEVSR | K3(Acetyl) | 437 | 678.05768 | 692.05768 | 3 |
| 27 | 438-452 | K*VPQVSTPTLVEVSR | K1(Acetyl) | 438 | 820.48047 | 841.48047 | 2 |
| 28 | 453-460 | NLGK*VGSK | K4(Acetyl) | 456 | 401.74799 | 422.74799 | 2 |
| 29 | 457-468 | VGSK*CCKHPEAK | K4(Acetyl) | 460 | 448.56024 | 462.56024 | 3 |
| 30 | 461-469 | CCKHPEAK*R | K8(Acetyl) | 468 | 395.86243 | 409.86243 | 3 |
| 31 | 469-496 | RMPCAEDYLSVVLNQLCVLHEK*TPVSDR | K22(Acetyl) | 490 | 818.91516 | 829.41516 | 4 |
| 32 | 546-558 | QIK*KQTALVELVK | K3(Acetyl) | 548 | 499.98828 | 513.98828 | 3 |
| 33 | 546-558 | QIKK*QTALVELVK | K4(Acetyl) | 549 | 749.47986 | 770.47986 | 2 |
| 34 | 549-562 | KQTALVELVK*HK*PK | K10(Acetyl), K12(Acetyl) | 558,560 | 810.0097 | 852.0097 | 2 |
| 35 | 563-581 | ATKEQLK*AVMDDFAAFVEK | K7(Acetyl) | 569 | 714.37738 | 728.37738 | 3 |
| 36 | 582-597 | CCK*ADDKETCFAEEGK | K3(Acetyl) | 584 | 917.38239 | 938.38239 | 2 |
| 37 | 585-598 | ADDK*ETCFAEEGKK | K4(Acetyl) | 588 | 524.24432 | 538.24432 | 3 |
| 38 | 585-598 | ADDKETCFAEEGK*K | K13(Acetyl) | 597 | 785.86145 | 806.86145 | 2 |
| 39 | 598-609 | K*LVAASQAALGL | K1(Acetyl) | 598 | 571.35791 | 592.35791 | 2 |

The asterisk * indicates the modified lysine

**References**

Morton, C.L., Potter, P.M. (2000). Comparison of Escherichia coli, Saccharomyces cerevisiae, Pichia pastoris, Spodoptera frugiperda, and COS7 cells for recombinant gene expression. Application to a rabbit liver carboxylesterase. *Mol Biotechnol*. 16, 193-202. doi: 10.1385/MB:16:3:193.

Lamego, J., Cunha, B., Peixoto, C., Sousa, M.F., Alves, P.M., Simplício, A.L., et al. (2013). Carboxylesterase 2 production and characterization in human cells: new insights into enzyme oligomerization and activity. *Appl Microbiol Biotechnol*. 97, 1161-1173. doi: 10.1007/s00253-012-3994-3.

Hu, Q., Tian, Z.H., Wang, H.N., Huang, J., Wang, FY., Zhao, B., (2021). Rational design and development of a novel and highly specific near-infrared fluorogenic substrate for sensing and imaging of human pancreatic lipase in living systems. *Sensors and Actuators*. 341, 130033. doi: 10.1016/j.snb.2021.130033

Giansanti, P., Tsiatsiani, L., Low, T.Y., Heck, A.J. (2016). Six alternative proteases for mass spectrometry-based proteomics beyond trypsin. *Nat Protoc*. 11, 993-1006. doi: 10.1038/nprot.2016.057.
